# A *Toxoplasma gondii* patatin-like phospholipase contributes to host cell invasion

**DOI:** 10.1371/journal.ppat.1008650

**Published:** 2020-07-06

**Authors:** Sarah K. Wilson, Justine Heckendorn, Bruno Martorelli Di Genova, Lindsey L. Koch, Peggy J. Rooney, Naomi Morrissette, Maryse Lebrun, Laura J. Knoll

**Affiliations:** 1 Department of Medical Microbiology and Immunology, University of Wisconsin—Madison, Linden Drive, Madison, Wisconsin, United States of America; 2 INSERM, Université Montpellier, Montpellier, France; 3 Stratatech Corporation, Charmany Drive, Madison, Wisconsin, United States of America; 4 Department of Molecular Biology and Biochemistry, University of California Irvine, Irvine, California, United States of America; Francis Crick Institute, UNITED KINGDOM

## Abstract

*Toxoplasma gondii* is an obligate intracellular parasite that can invade any nucleated cell of any warm-blooded animal. In a previous screen to identify virulence determinants, disruption of gene TgME49_305140 generated a *T*. *gondii* mutant that could not establish a chronic infection in mice. The protein product of TgME49_305140, here named TgPL3, is a 277 kDa protein with a patatin-like phospholipase (PLP) domain and a microtubule binding domain. Antibodies generated against TgPL3 show that it is localized to the apical cap. Using a rapid selection FACS-based CRISPR/Cas-9 method, a TgPL3 deletion strain (ΔTgPL3) was generated. ΔTgPL3 parasites have defects in host cell invasion, which may be caused by reduced rhoptry secretion. We generated complementation clones with either wild type TgPL3 or an active site mutation in the PLP domain by converting the catalytic serine to an alanine, ΔTgPL3::TgPL3^S1409A^ (S1409A). Complementation of ΔTgPL3 with wild type TgPL3 restored all phenotypes, while S1409A did not, suggesting that phospholipase activity is necessary for these phenotypes. ΔTgPL3 and S1409A parasites are also virtually avirulent *in vivo* but induce a robust antibody response. Vaccination with ΔTgPL3 and S1409A parasites protected mice against subsequent challenge with a lethal dose of Type I *T*. *gondii* parasites, making ΔTgPL3 a compelling vaccine candidate. These results demonstrate that TgPL3 has a role in rhoptry secretion, host cell invasion and survival of *T*. *gondii* during acute mouse infection.

## Author summary

*Toxoplasma gondii* is a eukaryotic parasite commonly found in warm-blooded animals worldwide. Successful replication of *T*. *gondii* within the host relies on its sophisticated cell invasion mechanism. Previous studies found that disruption of the *T*. *gondii* gene TgME49_305140 generated a mutant that could not establish a chronic infection in mice. The protein product of this gene, named TgPL3, is large with both patatin-like phospholipase and microtubule binding domains. Here we show that TgPL3 is localized to the apical end of *T*. *gondii*, which is used for invasion. A mutant *T*. *gondii* strain with a deletion of TgPL3 (ΔTgPL3) was generated. ΔTgPL3 parasites are defective for host cell invasion and do not cause disease in mice, even at high doses. Complementation of the TgPL3 gene back into ΔTgPL3 parasites repairs the invasion and mice infection defects. Moreover, complementation of ΔTgPL3 with a point mutation in the active site of the phospholipase domain (S1409A) did not rescue the invasion and mouse infection defects, suggesting the PLP domain is responsible for these phenotypes. Vaccination of mice with ΔTgPL3 and S1409A parasites protected them against lethal challenge with *T*. *gondii*, highlighting the potential of ΔTgPL3 as a vaccine strain.

## Introduction

*Toxoplasma gondii* is an obligate intracellular parasite that can infect all warm-blooded animals and any nucleated cell within the host. *T*. *gondii* is a member of the Apicomplexa phylum, which includes *Plasmodium spp*., the causative agent of malaria, and *Cryptosporidium*, one of the leading causes of waterborne disease outbreaks. Worldwide, *T*. *gondii* infects an estimated 30–50% of humans [1], which are dead-end intermediate hosts. The sexual cycle of *T*. *gondii* is restricted to the feline intestinal epithelium, from which *T*. *gondii* is excreted as oocysts in feces. The asexual cycle of *T*. *gondii* can occur in any warm-blooded animal and has two developmental stages: a rapidly replicating form called the tachyzoite and a slow growing stage called the bradyzoite. *T*. *gondii* is acquired orally either by ingestion of oocyst-contaminated vegetables or other foods, or by eating bradyzoite cyst-harboring meat products. Upon infection of an intermediate host, cyst and oocyst stages differentiate into the tachyzoite form and disseminate throughout the host before transitioning to the bradyzoite form. Bradyzoite-containing cysts reside in the brain and muscle tissue, and represent the long-term chronic phase of infection. Most infections are asymptomatic, but immunocompromised persons are at risk for severe primary infections or reactivation of a chronic infection that can lead to encephalitis or death. To date, no medications are available that can clear the chronic stage of *T*. *gondii*.

The *T*. *gondii* lytic cycle begins with a complex invasion mechanism. Gliding motility propels the parasite to find permissive host membranes [2]. Upon initial attachment, a tighter interaction is enabled by the formation of the moving junction (MJ). The MJ is a complex of the microneme protein apical membrane antigen 1 (AMA1) and rhoptry neck (RON) proteins, which are secreted from apical parasite organelles called micronemes and rhoptries, respectively [3]. While the RON proteins are typically part of the invasion machinery, the rhoptry bulb (ROP) proteins secreted during this process can modulate the host immune and metabolic pathways to be more favorable for parasite survival [4]. The parasite moves through the MJ, invaginating the host cell membrane to create a parasitophorous vacuole in which the parasite will replicate.

Previously, a signature tagged mutagenesis (STM) screen identified insertional *T*. *gondii* mutants that showed a decrease in chronic cyst burden compared to wild type parasites *in vivo* [5]. One of the identified mutants corresponded to TgME49_305140, annotated as a patatin-like phospholipase (PLP) domain containing protein, and reported here as TgPL3. PLP enzymes were first discovered in potato tubers and played a role in storage [6, 7], but these enzymes have since been implicated in both lipid metabolism and inflammation in plants, animals, and more recently microorganisms. Bacterial PLPs have phospholipase activity and are virulence factors [8, 9]. There are many PLP domain containing proteins across the Apicomplexa phylum and six putative PLP enzymes in *T*. *gondii*, two of which have been investigated [8]. The first described *T*. *gondii* PLP, named TgPL1, is critical for survival of parasites in activated macrophages [10]. During macrophage activation and bradyzoite cyst development, TgPL1 changes location from within punctate vesicles in the cytoplasm to the parasitophorous vacuole membrane [11, 12]. In late chronic infection, mice infected with ΔTgPL1 parasites had increased expression of inflammatory cytokines and fewer brain lesions from reactivated cysts compared to wildtype-infected mice [13]. Another *T*. *gondii* PLP, TgPL2, was shown to localize to the apicoplast and play a critical role in lipid homeostasis within that organelle [14]. Knockdown of TgPL2 led to excessive accumulation of membranes around the apicoplast, altered the lipid profile of the parasite, and resulted in parasite death over time.

Here we present the functional characterization of TgPL3. Along with the PLP domain, TgPL3 contains a microtubule binding domain and is localized in the apical end of the parasite. ΔTgPL3 parasites have reduced host cell invasion, which may be caused by a defect in rhoptry secretion. ΔTgPL3 parasites are also virtually avirulent *in vivo*, with reduced cytokine stimulation and parasitemia. Mutation of the active site serine recapitulated the *in vivo* and *in vitro* defects, suggesting that phospholipase activity is responsible for rhoptry secretion and invasion. These results show that TgPL3 plays a role in invasion of host cells, unique from the first two characterized PLPs.

## Results

### TgPL3 has predicted microtubule binding and patatin-like phospholipase domains

**Fig 1:**
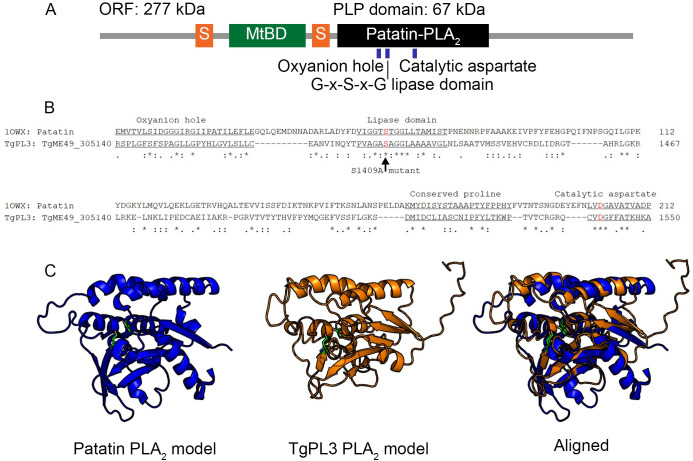
Predicted domains of TgPL3 based on sequence and structural alignments. **(A)** In orange, two serine-rich domains (S) flank the microtubule binding domain (MtBD) in green. The first serine-rich domain contains 34 consecutive serines and the second contains 37 serines interrupted by 2 prolines and 2 alanines. The putative PLP domain is shown in black. **(B)** The PLP domain contains all the necessary conserved amino acids for activity based on sequence alignment. The known conserved patatin motifs are underlined and catalytic S/A dyad are colored red. The serine to alanine mutation (S1409A) is indicated by an arrow. **(C)** Structural alignment of the TgPL3 PLP domain (orange) to the patatin PLA_2_ crystal structure (blue) predicts correct folding to form the active site. The secondary and tertiary structures of the TgPL3 PLP domain were predicted using I-TASSER and the resulting model was aligned to the PDB model of patatin (1OWX) in PyMOL. The catalytic serine and aspartate dyad are shown in green in each model.

Sequence and structural alignment of the 277kDa TgPL3 protein revealed a PLP domain predicted to have phospholipase A_2_ (PLA_2_) activity and a MIP-T3 microtubule-binding domain (MtBD) surrounded by polyserine stretches (Fig 1A), which may act as flexible linkers allowing the binding domain to be accessible to its target [15]. PLP domains include a glycine-rich oxyanion hole, a catalytic serine-aspartate dyad where the serine falls within the lipase motif G-X-S-X-G and a conserved proline between the serine and aspartate (Fig 1B). Humans have nine predicted PLPs and all of these PLPs contain the catalytic serine-aspartate dyad [16]. To further analyze the PLP potential of TgPL3, we threaded the protein sequence of its PLP domain onto the crystal structure for the plant PLP [17]. The TgPL3 PLP domain aligns with the plant model to form an active site (Fig 1C).

### TgPL3 localizes to the apical end of *T*. *gondii*

**Fig 2:**
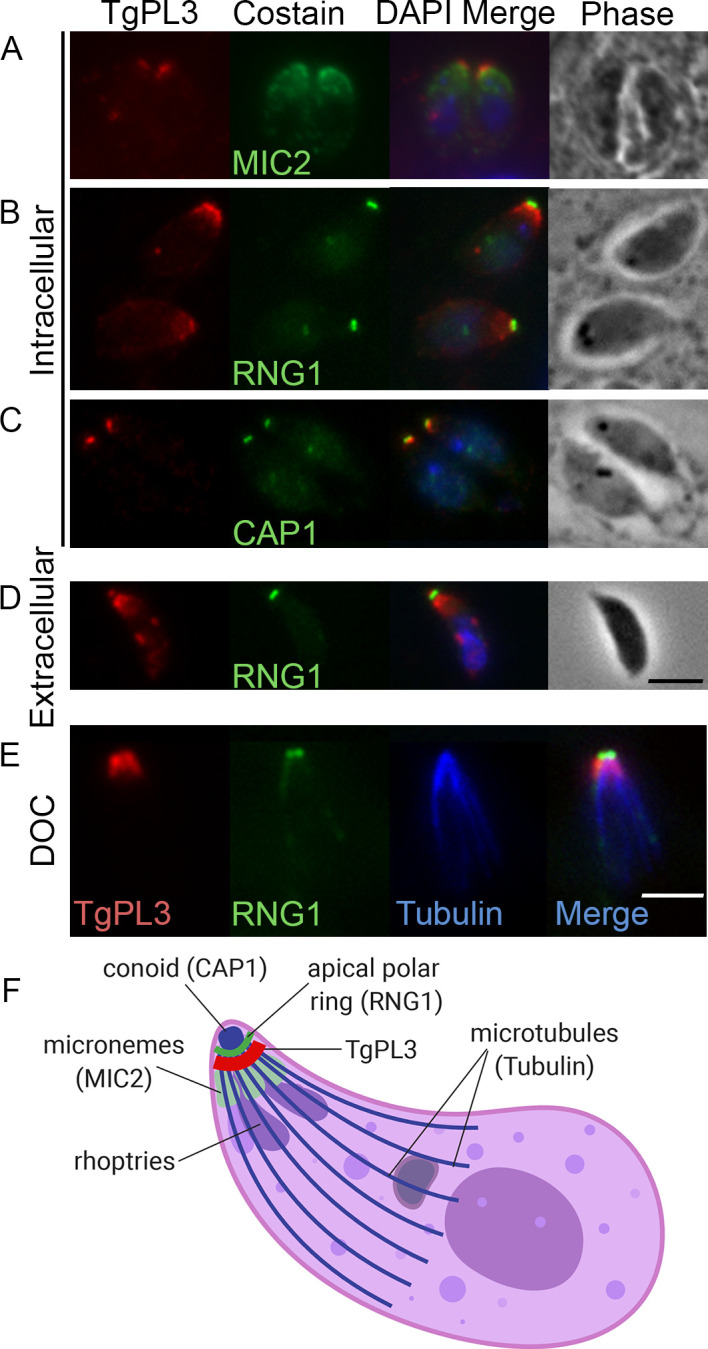
TgPL3 localizes to the apical end of tachyzoites. Top three panels show immunofluorescence of TgPL3 (red) in intracellular tachyzoites co labeled with **(A)** MIC2, **(B)** RNG1 or **(C)** CAP1 (green). Bottom two panels show TgPL3 localization in extracellular tachyzoites after **(D)** induced conoid extrusion or **(E)** deoxycholate extraction (DOC). **(F)** Schematic showing costain localization in intracellular parasites (created with BioRender.com). Images for panels A, B, C and D were taken under the same magnification and the scale bars in the lower right corners equal 5 μm.

The previous two PLP genes characterized in *T*. *gondii* had unique localizations and functions. To localize TgPL3, we generated antibodies against TgPL3 by immunizing mice with either a wheatgerm-purified PLP domain (Fig 2) or three different synthesized peptides (S1A Fig). All antibodies generated showed TgPL3 localizing to the apical end of intracellular RHΔKu80ΔHPT parasites. Counter staining with MIC2 antibodies revealed TgPL3 localizes above the micronemes (Fig 2A, S1B). We next examined the apical ring and conoid regions further using RNG1-YFP and CAP1-YFP fusion RH *T*. *gondii* strains [18,19]. In intracellular parasites, TgPL3 localizes below both TgRNG1 and TgCAP1 (Fig 2B & 2C). However, in extracellular parasites where conoid extrusion is induced, TgPL3 partially extends out past the apical ring along with the conoid (Fig 2D).

As described above, TgPL3 has a predicted microtubule-binding domain (Fig 1A). To examine cytoskeleton association, we extracted the cytoskeleton of extracellular parasites in deoxycholate (DOC) detergent. When *T*. *gondii* are lysed with DOC, only the cytoskeleton and its tightly bound proteins remain [18]. After DOC extraction, TgPL3 colocalized to the apical end, identified using counterstaining for ß-tubulin (Fig 2E and S2 Fig). Thus, TgPL3 is localized to the apical cap region of the parasite (schematized in Fig 2F).

### Purification of the TgPL3 patatin-like domain and testing for PLA_2_ activity

To test if TgPL3 had PLA_2_ activity we first purified the 67 kDa PLP domain. Attempts to express the PLP domain in *E*. *coli* were unsuccessful, likely due to the toxicity of the protein in live cells. We then switched to an in vitro transcription and translation system to express the TgPL3 PLP domain. The domain was successfully expressed in wheat germ extract; however, multiple attempts to show PLA_2_ activity using a thin layer chromatography assay were unsuccessful. It is likely that expressing the 67 kDa PLP domain apart from the rest of the 277kDa TgPL3 protein creates a misfolded protein or otherwise inactive protein. For example, the *Pseudomonas aeruginosa* PLP requires ubiquitin or ubiquitinated proteins as cofactor for in vitro activity [20]. To address potential PLA_2_ activity for this manuscript, we engineered a TgPL3 complementation construct with TgPL3 containing a single point mutation that changes the predicted catalytic serine in the lipase domain to an alanine (Fig 1B).

### Rapid fluorescence activated cells sorting (FACS) needed to obtain TgPL3 deletion

The TgPL3 mutant discovered in the STM screen [5] contained an insertion in the TgME49_305140 promoter that created a fusion transcript of the chloramphenicol acetyl transferase (CAT) and TgPL3 coding regions controlled by the constitutively active α-tubulin promoter (S3 Fig). To assess the role of TgPL3 in pathogenesis, we deleted the entire open reading frame (ORF) in RHΔKu80ΔHPT parasites by CRISPR. Initial attempts to delete the TgPL3 ORF from *T*. *gondii* strain ME49ΔHPT using hypoxanthine-xanthine-guanine phosphoribosyl transferase (HPT) as the positive selectable marker and uracil phosphoribosyl transferase (UPT) as the negative selectable marker were unsuccessful. While PCR indicated a population of successful TgPL3 deletion mutants in newly transfected parasites, these deletion mutants were rapidly outcompeted by drug-resistant random insertion mutants during drug selection. The recent genome-wide CRISPR screening database revealed this gene has a phenotype score of -3.48, which predicts that this gene is fitness conferring during infection of human fibroblasts [21].

**Fig 3:**
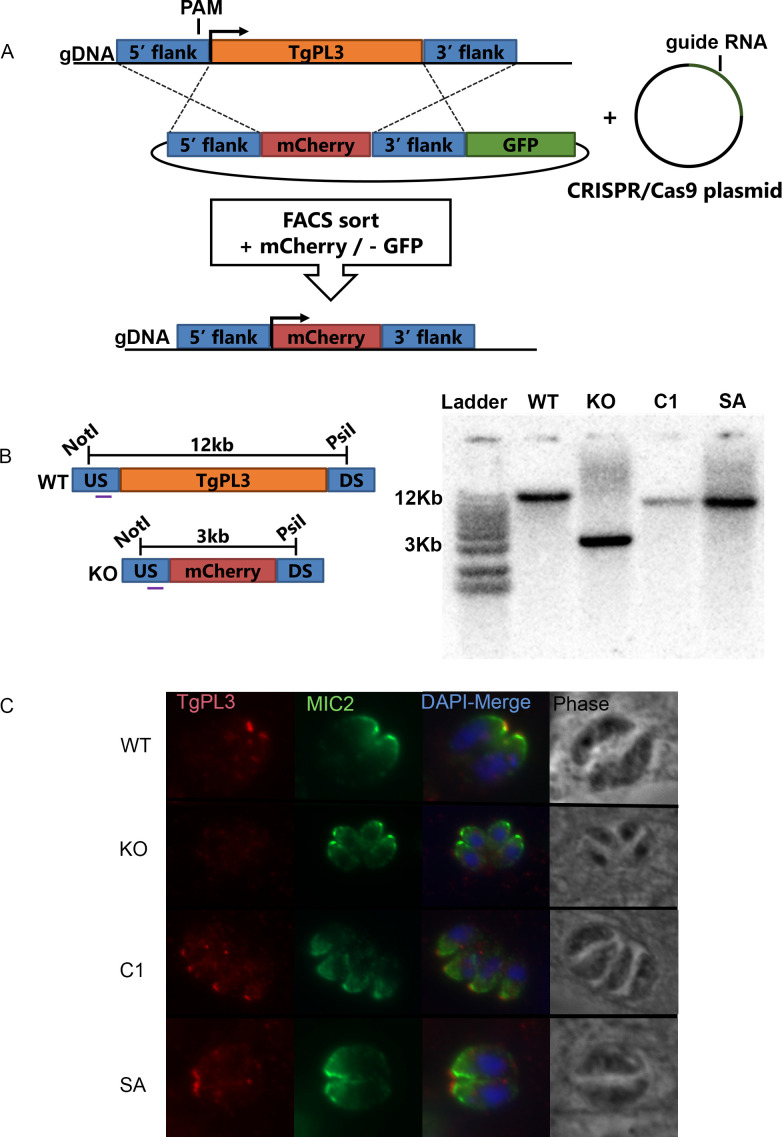
Deletion of TgPL3 from RHΔKu80 parasites using FACS sorting. **(A)** Schematic for the targeted knockout of the TgPL3 gene in *T*. *gondii* by double homologous crossover. Parental strain RHΔku80ΔHXGPRT parasites were electroporated with the CRISPR/Cas9 plasmid containing gRNA targeting the 5’ end of TgPL3 and the knockout plasmid containing TgPL3 5’ and 3’ flanking regions surrounding mCherry which allows for sorting mCherry positive parasites using FACS. Sorting GFP negative clones in tandem ensures a double crossover event. **(B)** Genomic DNA of clones were screened by southern blot using a probe targeting the 5’ upstream (US) flanking region of TgPL3. Parental RHΔku80ΔHXGPRT (WT), complement (C1) and S1409A (SA) parasites show a 12kb band, ΔTgPL3 (KO) strains show a 3kb band. **(C)** Parasite strains were further analyzed by immunofluorescence of TgPL3 (red) co-labeled with MIC2 (green). Images were taken under the same magnification and the scale bar in the lower right corner equals 5 μm.

Therefore, we created a rapid screening process using fluorescent markers in place of drug-selectable markers. This method uses mCherry as a positive marker and GFP as a negative marker (Fig 3A). After transfection, the parasites were allowed to invade and replicate for 2–3 days without serial passage, whereupon they were sorted by flow cytometry. Parasites that were mCherry positive and GFP negative were sorted directly into 96-well plates for clone isolation. In addition, the RHΔKu80ΔHPT *T*. *gondii* strain, which lack the non-homologous end-joining protein Ku80, was used for knockout generation to virtually eliminate random insertion of the DNA construct [22]. After transfection into the RHΔKu80ΔHPT strain, we saw mCherry positive and GFP negative populations during FACS (S4 Fig), whereas transfection into to ME49ΔHPT parasites yielded primarily random insertion mutants rather than knockout clones. We isolated several TgPL3 deletion (ΔTgPL3) clones from the RHΔKu80ΔHPT transfection. These clones were confirmed to be TgPL3 deletions by Southern blot using a probe specific to the 5’ flanking region of TgME49_305140, which was present in both the endogenous and knockout loci (Fig 3B). We also confirmed the deletion by immunofluorescence assay using the anti-TgPL3 antibody and found that TgPL3 was present in the apical end in parental parasites but not the ΔTgPL3 mutants (Fig 3C).

### Complementation of ΔTgPL3 parasites

To ensure the *in vitro* phenotypes associated with loss of TgPL3 were not due to CRISPR off-target effects, we complemented the ΔTgPL3 parasites. We generated ΔTgPL3::TgPL3 clones (complement) by targeting *TgPL3* cDNA back into the endogenous locus using a CRISPR/Cas9 plasmid with a mCherry specific guide RNA and screened for loss of mCherry expression. We also created a ΔTgPL3::TgPL3^S1409A^ complement strain (S1409A) with an active site mutation in the PLP domain by converting the catalytic serine to an alanine (Fig 1C). Complement clones were sorted by flow cytometry and screened by southern blot (Fig 3B). Restoration of TgPL3 protein was confirmed by immunofluorescence assay (Fig 3C).

### ΔTgPL3 parasites have an invasion but not an attachment defect

**Fig 4:**
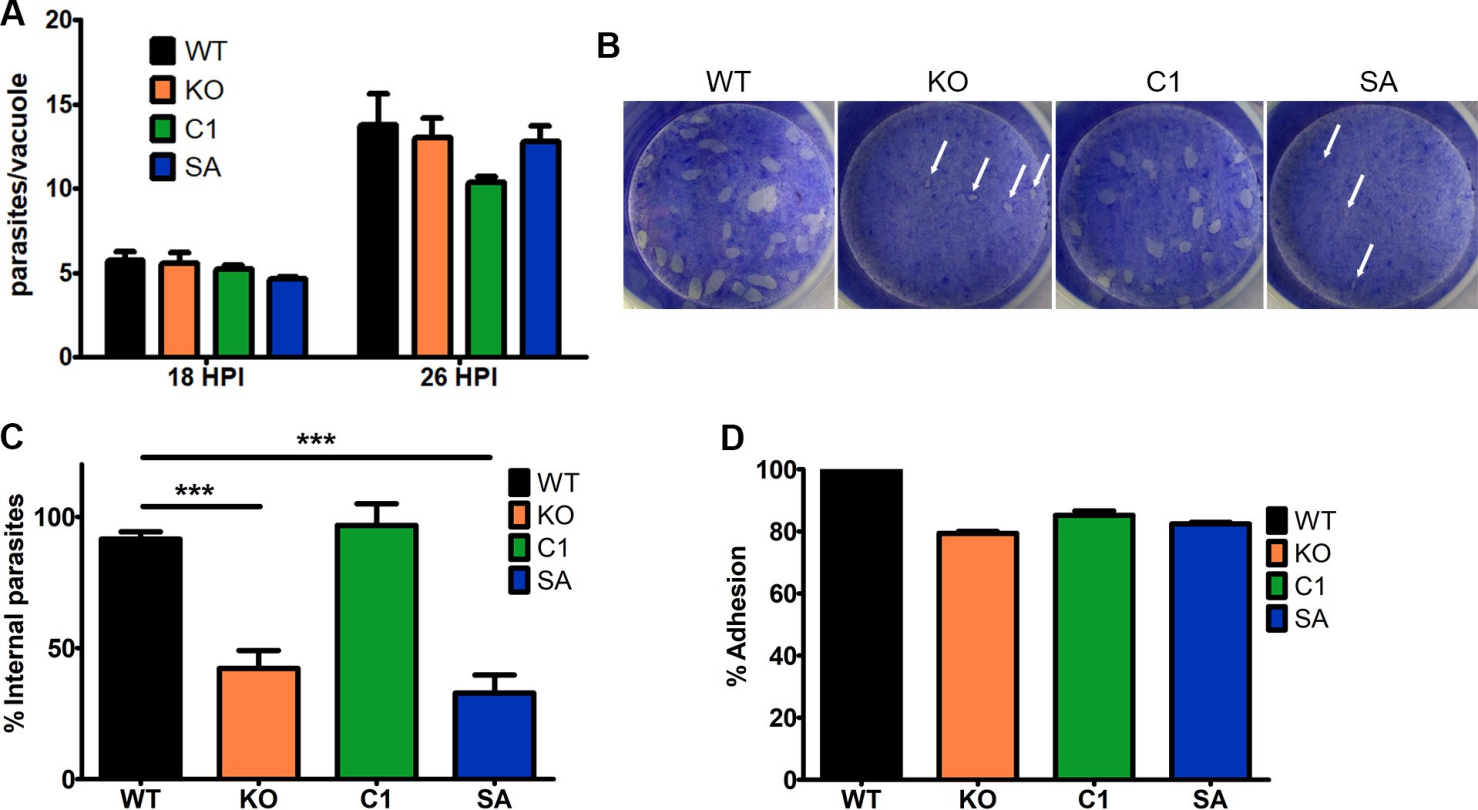
TgPL3 is critical for invasion but not attachment. **(A)** Triplicate monolayers of HFFs were infected with 3.4 x 10^4^ *T*. *gondii* parasites. At 18 or 26 hours post infection, tachyzoites per vacuole were scored in at least 100 randomly encountered vacuoles per replicate. Average tachyzoites per vacuole is shown at each time point. P value was nonsignificant in a two tailed t-test compared to WT. **(B)** 9 days post infection, HFF monolayers were stained with crystal violet to reveal *T*. *gondii* plaque formation. White arrows indicate the small plaques created by the KO and SA parasites. **(C)** The percentage of parasites that were successfully able to invade the host cell was determined using the red/green invasion assay [21]. Four independent experiments were performed in triplet, and the combined results are shown here. *** p<0.0001. **(D)** The percentage of parasites that were attached to glutaraldehyde fixed host cells was examined but no significant differences were seen. Shown here the combined results from two independent experiments performed in triplet.

The difficulty obtaining ΔTgPL3 parasites and a -3.48 CRISPR score [21] suggested there would be a severe growth defect with the loss of TgPL3. Indeed, we regularly passaged more ΔTgPL3 parasites than parental strain to maintain synchronized cultures. We first analyzed the internal replication rate of each strain by counting the number of parasites per vacuole at 18 and 26 hours post-invasion. We did not observe significant differences in the replication rates of invaded parasites between the parental, knockout and complement strains (Fig 4A and S5 Fig). However, plaque assay, which measures parasite growth over multiple lytic cycles, showed fewer and smaller plaques in ΔTgPL3 and S1409A compared to WT and complement strains (Fig 4B and S6A Fig). The localization of TgPL3 to the apical end of the parasite suggested involvement in invasion, so we then evaluated the ability of the mutants to invade using the red/green invasion assay [23]. The parasites were allowed to infect fibroblasts for 5 minutes prior to fixation and extracellular and intracellular parasite were quantified by differential staining. A significantly lower percent of ΔTgPL3 parasites invaded the host cell compared to the parental strain (Fig 4C and S7 Fig). This invasion defect was restored by complementation with TgPL3 but not S1409A, highlighting that phospholipase activity is likely involved in the invasion phenotype. Because the invasion assay is biased based on the ability of the parasite to successfully attach prior to invasion, we analyzed the ability of the parasites to attach using the glutaraldehyde fixed host cell assay [24]. We saw no difference in the attachment between the parental, knockout and complement strains (Fig 4D and S8 Fig). As a negative control we incubated all parasites strains with BAPTA-AM and saw no parasite attachment (S8 Fig). Finally, when we induced the egress of intracellular parasites, there was no difference in the ability of each strain to successfully egress from the host cell (S6B Fig).

### Invasion defect of ΔTgPL3 is likely linked with reduced rhoptry and not microneme secretion

**Fig 5:**
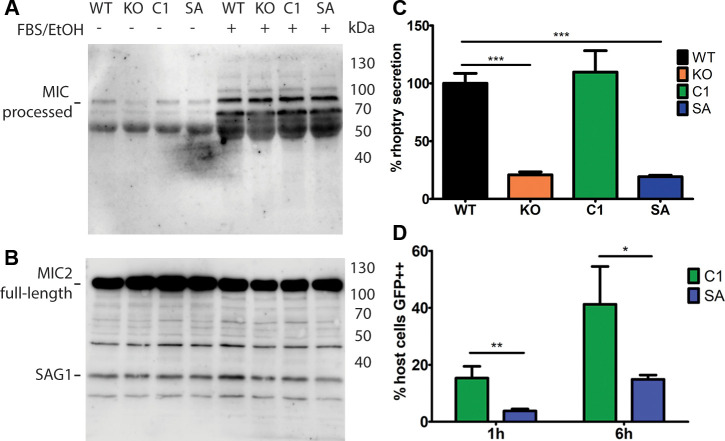
TgPL3 enhances rhoptry but not microneme secretion. Excreted/secreted antigen (ESA) was induced in 1 x 10^8^ parasites by the addition of 3% fetal bovine serum and 1% ethanol (FBS/EtOH). Supernatant **(A)** and pellet **(B)** were run on a 12% SDS-page gel, transferred to PVDF membranes and simultaneously probed with rabbit anti-MIC2 and mouse anti-SAG (monoclonal DG52). **(A)** Western blotting showing processed MIC-2 (95–100 kDa) and no SAG1. Size markers are indicated on the right side in kDa. **(B)** Pellet or loading control shows the full-size MIC-2 band (115 kDa) and a lower band corresponding to SAG1 (30 kDa). **(C)** Quantification of rhoptry discharge during e-vacuole formation. Triplicate monolayers of HFFs were infected with 5x10^6^ parasites that were pre-treated with cytochalasin D. After a 15-minute incubation, the parasites were stained with a mouse α-SAG1 antibody and a rabbit α-ROP1 antibody. After counting, percent rhoptry secretion was normalized to the WT strain. Three independent experiments were performed, and the combined results are shown here. *** p<0.001 **(D)** Rhoptry secretion by the SeCreEt assay [28] and the number of GFP++ host cells was quantified by FACS analysis, as shown on the y-axis. After 1 hour of invasion, ΔTgPL3 parasites complemented with S1409A showed a significant difference in rhoptry secretion compared to parasites complemented with the WT gene (** p<0.01). After 6 hours, this difference was less significant (* p<0.05). Shown here the results from one of two independent experiments performed in triplet.

Invasion involves sequential secretion from micronemes and rhoptries. Therefore, to further investigate the mechanism behind the invasion defect of ΔTgPL3, we analyzed the ability of the parasites to discharge the content of both organelles. We induced the secretion of microneme proteins AMA1 and MIC2 by either ethanol and serum [25] or propranolol, and then monitored protein secretion by western blot. We saw no apparent difference in the ability of each strain to secrete AMA1 or MIC2 (Fig 5A, 5B and S6B, S6C Fig). We also determined rhoptry secretion in cytochalasin D (CytD) treated parasites, which inhibits actin polymerization and blocks invasion [26]. During invasion, rhoptry proteins are injected into the host cell and in CytD-treated parasites, clusters of ROP1-positive vesicles, called evacuoles, are visible inside the host cell [27]. We evaluated the formation of evacuoles in the different parasite lines and found that the ΔTgPL3 and S1409A parasites were significantly reduced in rhoptry section (Fig 5C and S9 Fig).

We also determined rhoptry secretion by the SeCreEt assay, which uses rhoptry protein toxofilin coupled to Cre-recombinase to induce GFP expression in DsRed host cells [28]. The complement (C1) and S1409A (SA) parasites were allowed to invade the host cells for either 1 or 6 hours before changing the media to remove any remaining external parasites. After 24 hours, infected cells were trypsinized and the percentage of GFP expressing cells (successful rhoptry secretion) was determined by FACS. With 1 hour of invasion, ΔTgPL3 parasites complemented with the WT gene had significantly more GFP positive host cells than the ΔTgPL3 parasites complemented with the S1409A mutant gene (p<0.01, Fig 5D and S10 and S11 Figs). With 6 hours of invasion, this difference was less significant (p<0.05) in the first experiment, and not significant in the second experiment, highlighting that ΔTgPL3 parasites may be delayed in rhoptry secretion and will “catch-up” overtime in tissue culture. All together, these results suggest that a role of TgPL3 during invasion is specific to rhoptry secretion.

### ΔTgPL3 and S1409A parasites are avirulent *in vivo* and provide protection against subsequent infection

**Fig 6:**
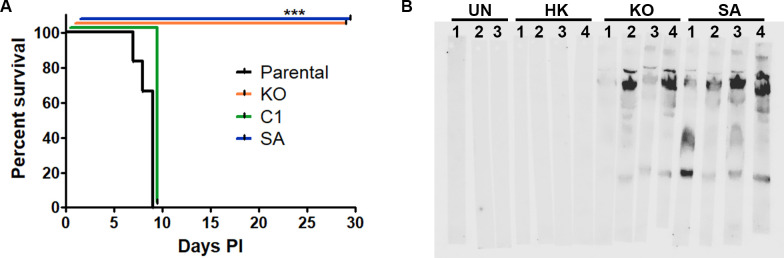
ΔTgPL3 and S1409A parasites are avirulent. **(A)** Shown is a combination of two independent experiments of 3–4 NMRI mice each, either all male or all female, with a total of 7 mice per strain. Mice were infected with 1000 *T*. *gondii* parasites and survival was monitored for 28 days post infection. No brain cysts were seen in surviving mice. *** p<0.001. **(B)** Serum collected 28 days post infection in surviving mice tested for seropositivity with individual strip blots for each mouse (labeled 1, 2, 3 or 4). Seroconversion was seen in all ΔTgPL3 and S1409A infected mice but not in uninfected mice (UN) or mice infected with heat-killed parasites (HK). Shown is a representative of two independent experiments.

We then tested if the apparent defect in rhoptry secretion of the ΔTgPL3 and S1409A mutant parasites would affect their virulence in a mouse model. ΔTgPL3 and S1409A complemented strains did not cause any disease symptoms in mice, even when infected with up to 1x10^5^ parasites. The parental RHΔKu80ΔHPT strain is a virulent type I strain, and infection with a dose of 100 parental or complement parasites resulted in all mice succumbing to infection by day 11 (S12A Fig). Mice infected with a 1000 parental or complement parasites succumbed by day 9 (Fig 6A). All mice infected with 100, 1000, 1x10^4^ and 1x10^5^ ΔTgPL3 and S1409A mutant parasites survived acute infection and did not show signs of disease (Fig 6A, and S12A, S14 Figs). At 28 days post infection, brains were analyzed for bradyzoite cysts by staining with Dolichos biflorus agglutinin (DBA) and sera was tested for parasite antibody production. No cysts were found in mice infected with either ΔTgPL3 and S1409A parasites (S13 Fig), but all mice seroconverted (Fig 6B, S14 Fig). These results suggest immune exposure, but that these mutants were unable to disseminate and/or establish chronic infection. Mice infected with 1000 heat-killed parental parasites did not seroconvert, so the immune responses seen against ΔTgPL3 and S1409A infection were not simply a reaction to non-viable parasites (Fig 6B).

**Fig 7:**
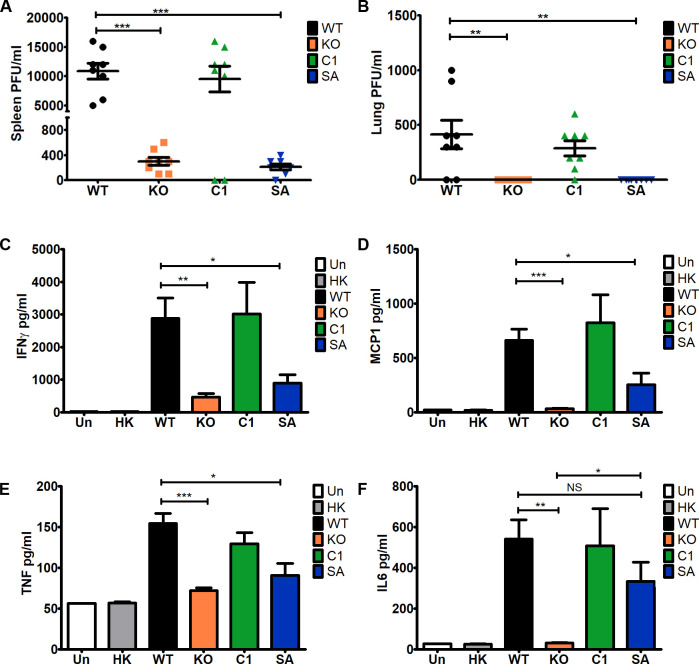
ΔTgPL3 and S1409 parasites are unable to disseminate *in vivo*. In 2 independent experiments, 4 male NMRI mice were infected with 1000 *T*. *gondii* parasites. 3 days post infection **(A)** spleens and **(B)** lungs were removed and homogenized. Homogenate was serially diluted, added to HFF monolayers in duplicate, and plaqued for 7 days. The monolayers were stained with crystal violet and PFU/ml calculated. **(C-F)** Serum was collected from the same mice at 3 days post infection and cytokine levels of **(C)** IFNγ, **(D)** MCP1, **(E)** TNF and **(F)** IL6 were determined using a cytokine bead array kit (Biosciences). Cytokine analysis was repeated with female mice and shown is one representative experiment. Uninfected mice and mice infected with 1000 heat-killed parasites were used as negative controls * p<0.05, **p <0.01 and ***p<0.001.

To determine whether ΔTgPL3 and S1409A parasites were viable during infection and capable of dissemination, we performed a plaque assay on the lungs and spleen of mice at day 3 post infection. After infection with 1000 parasites, the parental and complement strains had high parasite infection in both organs, whereas ΔTgPL3 and S1409A mutant parasites had significantly fewer parasites present in the spleen and no parasites in the lung (Fig 7A and 7B). While both ΔTgPL3 and S1409A mutant parasites induced a significantly lower serum cytokine response than the parental and complement strains, they did induce a higher response than that of either uninfected mice or mice injected with heat-killed parasites (Fig 7C–7F and S12B–S12E Fig). These results suggest that ΔTgPL3 and S1409A mutant parasites were capable of low levels of replication in mice at or near the site of injection, but that they were unable to disseminate beyond the peritoneal cavity.

**Fig 8:**
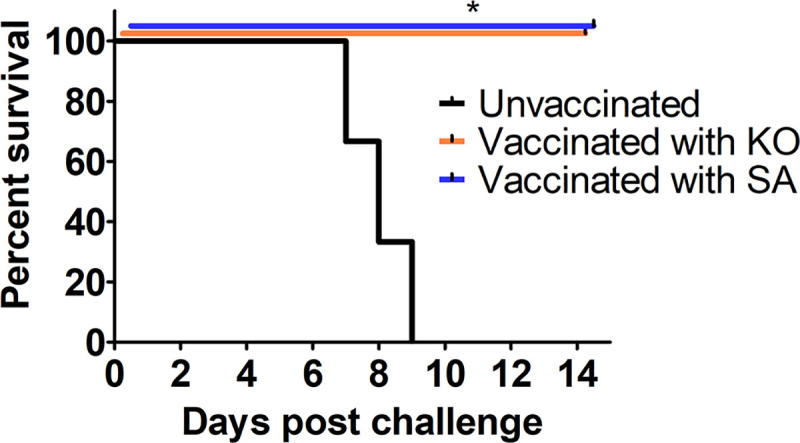
ΔTgPL3 and S1409 parasites induce a protective immunity against subsequent infections. 3 male NMRI mice were infected with 1000 ΔTgPL3 or S1409 parasites, or uninfected as a negative control. 30 days post infection, mice were tested for seropositivity and challenged with 1000 RHΔKu80ΔHPT parasites. Survival was recorded for 14 days post challenge.

The vaccine potential of ΔTgPL3 and S1409A parasites to form protective immunity against subsequent infection was assessed. Mice were vaccinated with 1000 ΔTgPL3 or S1409A parasites and at 30 days post infection, serum samples were collected to confirm seropositivity (S15A Fig). These vaccinated mice, as well as age-matched uninfected control mice, were then challenged with 1000 parental RHΔKu80ΔHPT parasites. Survival, disease symptoms, and weight change was recorded for 14 days post challenge. While all of the control mice succumbed to acute infection within 9 days, all vaccinated mice infected with ΔTgPL3 and S1409 survived the subsequent lethal challenge (Fig 8). These mice did show minor symptoms of disease, such as rough fur and weight loss. As early as 10 days post infection, they all appeared healthy and active again with no visible disease symptoms (S15B and S15C Fig).

## Discussion

Here we define the role of the third characterized *T*. *gondii* PLP, TgPL3, that is localized to the apical cap of the parasite. Cytoskeleton extraction of extracellular parasites with deoxycholate shows that TgPL3 likely binds to the cytoskeleton. TgPL3 was identified previously in a screen to identify genes involved in chronic cyst formation [5]. However, the insertion formed a fusion transcript with the *TgPL3* gene, prompting us to create a full knockout strain to allow assessment of the locus. To generate ΔTgPL3 parasites, we used a FACS-based cloning method that would reduce the opportunity for competition among a population of transfected parasites. By using fluorescent proteins to sort parasites rather than drug selectable markers, we cloned the mCherry+/GFP- parasites into individual wells without passage after transfection, which allowed us to obtain a mutant with a severe invasion defect. Finally, as *T*. *gondii* strains change their virulence after serial passage in tissue culture, flow cytometry without passage after transfection reduced the opportunity for parasites to evolve away from their parental strain. Deletion of TgPL3 showed multiple phenotypes including reduced rhoptry secretion and invasion, and a severe virulence defect. All of these defective phenotypes were restored in parasites complemented with the entire ORF, but not if it contained a single point mutation in the active site of the PLP domain, changing a serine to alanine. These results highlight that the phenotypes due to TgPL3 deletion are most likely due to the loss of phospholipase activity.

Knockout of TgPL3 resulted in an invasion defect that accumulated over several lytic cycles to form smaller and fewer plaques than the parental parasites. Because rhoptry neck proteins play a critical role for invasion by building the moving junction necessary for intimate attachment of the parasite to the host cell, the possible defect in rhoptry secretion could be causing the invasion defect seen. Additionally, mutation of the active site serine recapitulated the invasion defects, suggesting that phospholipase activity is required for rhoptry secretion. Gliding motility, microneme secretion, and egress were apparently unaffected by ΔTgPL3 or S1409A, so the role of TgPL3 is not ubiquitous during invasion.

PUFAs and lysophospholipids (LPLs) are cleaved from phospholipids by PLA_2_ and the release of these products destabilizes the membrane. Rhoptries have a higher membrane rigidity than the parasite as a whole [29], so PLP activity could have a significant effect on local membrane fluidity of the rhoptries, allowing for release of embedded rhoptry proteins in the organelle’s membranes. PUFAs and LPLs can be recycled back into the lipid membrane or serve as signaling molecules to regulate lipolysis, lipogenesis, and the immune responses [8]. Phospholipase C activity was previously shown to be involved in invasion signaling. The enzyme cleaves phosphatidyl inositol 4,5‐biphosphate to release IP3. When IP3 binds with its receptor IP3R, Ca^2+^ is released and triggers conoid extrusion, a critical step for successful invasion. [30]. TgPL3 could play a similar role in signaling for rhoptry secretion rather than a mechanical release of the proteins. Finally, PUFAs have been shown to act on syntaxin, a member of the soluble NSF-attachment receptor (SNARE) protein family involved in fusion of vesicles with the plasma membrane [31], so the generation of PUFAs through PLP activity could have a direct, essential action on the final stages of membrane fusion. We predict that the *in vivo* activity of TgPL3 is either involved in the mechanical release of the rhoptries themselves or its products are used as signaling molecules to induce the release of rhoptries. Future analysis will determine if and how the PLP activity plays a role during rhoptry secretion.

The tissue culture invasion defects conferred by the ΔTgPL3 were recapitulated when ΔTgPL3 was complemented with the entire TgPL3 ORF, but not if it contained a S1409A mutation. As TgPL3 is a large protein, most of which does not have predicted functional domains, we compared ΔTgPL3 and S1409A parasites in a mouse model to test whether there would be additional phenotypes of ΔTgPL3 *in vivo* that would be complemented by the S1409A construct. ΔTgPL3 and S1409A parasites were both virtually avirulent *in vivo*, with reduced cytokine stimulation, parasitemia and dissemination beyond the injection site. However, the S1409A mutant induced a statistically significant increase in IL6, and TNFα in the lower doses, *in vivo* compared to ΔTgPL3, suggesting a yet undefined role for TgPL3 beyond the PLP domain. IL-6^-/-^ mice are significantly more susceptible to *T*. *gondii* infection than WT mice due to a failure to produce a robust type I immune response during early infection, followed by a significantly amplified proinflammatory response during late acute and chronic infection that often proves lethal to the host [32]. Compared to WT mice, TNF^-/-^ mice fail to control *T*. *gondii* growth during chronic infection and succumb significantly earlier to encephalitis [33]. In future studies, truncated mutants that maintain PLP activity could determine alternative functions for TgPL3 that are outside of the PLP domain but currently being masked by the severe invasion defects.

Previously, the *T*. *gondii* armadillo-repeats-only protein (TgARO) was shown to be defective in rhoptry secretion [34]. Mice infected with ΔTgARO parasites survived acute infection and seroconverted similar to our mutants, but in this case, they failed to mount a protective immune response. Mice infected with the ΔTgPL3 and S1409A mutants not only survived acute infection, they never even showed the typical signs of disease such as ruffled fur or a humped back. However, these mice still produced a robust antibody response, which increased in a dose-dependent manner with the initial parasite infectious dose. Thirty days after initial infection with ΔTgPL3 and S1409A mutants, mice survived a secondary challenge with a lethal dose of WT parasites with minimal disease symptoms. These results suggest that TgPL3, or even the PLP specifically, could be a compelling target for livestock vaccination. Toxoplasmosis is estimated to cause 10–23% of spontaneous abortion in sheep and goats in Europe and the United States [35]. Vaccinating livestock with ΔTgPL3 or S1409A parasites could protect against future infections or reactivation of chronic infections and would likely be a benefit to the agriculture economy.

To better understand the mechanism behind the phenotypes we saw in tissue culture and *in vivo*, we purified the PLP domain of TgPL3 for *in vitro* activity assays. Because we were unable to express the PLP domain in *E*. *coli*, likely due to the toxicity of expressing a lipase in live cells, we changed to an *in vitro* transcription and translation system using wheat germ extract. Although the domain was successfully expressed, attempts to show activity using a thin layer chromatography assay have been unsuccessful. TgPL3 is a 2579 amino acid protein of which the expressed PLP domain is only 67 kDa. It is likely that the domain alone is inactive or unable to fold correctly. It is also possible that TgPL3 requires a cofactor, similar to the *Pseudomonas aeruginosa* PLP, ExoU, which requires host ubiquitination to activate the PLA_2_ activity [20], and the absence of this cofactor limits enzymatic activity even with proper folding. Future studies will address the substrate specificity of TgPL3 PLP domain to identify which specific phospholipids are targeted during rhoptry secretion.

PLP domains are present in high numbers across the Apicomplexa phylum, but none of these proteins have been characterized outside of *T*. *gondii*. None of the characterized PLP containing genes have shown activity *in vitro*, which could again indicate a need for additional host factors and further optimized assay buffers for parasite PLPs. Lipid metabolism and signaling is critical during the parasite lifecycle [36], but there is still much to discover about these complex processes. Future work on the remaining *T*. *gondii* PLPs and other parasite derived PLPs may reveal novel regulators of parasite replication, infection and virulence.

## Materials and methods

### Ethics statement

Mice were treated in compliance with the guidelines set by the Institutional Animal Care and Use Committee (IACUC) of the University of Wisconsin School of Medicine and Public Health (protocol #M005217). The institution adheres to the regulations and guidelines set by the National Research Council.

### Parasite and host cell culture

*Toxoplasma gondii* tachyzoites were cultured in human foreskin fibroblasts (HFF, ATCC) in Dulbecco’s modified Eagle’s medium (DMEM, Gibco) supplemented with 10% fetal bovine serum, 2mM L-glutamine, and 1% penicillin-streptomycin at 37°C with 5% CO_2_.

### Generation of TgPL3 knockout and complementation strains

Empty vector pBC_mCherry_GFP was created by digesting a pBC SK(-) vector with KpnI and PciI and inserting mCherry and GFP (mgfp5) PCR products using primers GFP GA F/R and mCherry GA F/R (S1 Table). The mCherry and GFP fragments were amplified from vectors optimized for expression in *T*. *gondii*, where both contain 5’ αTub promoters with 3’ DHFR and SAG1 UTRs, respectively. pBC_mCherry_GFP was digested with SpeI and KpnI to insert the flanking regions of our gene of interest on either side of the mCherry positive marker. 1kb genomic fragments 5’ and 3’ of the TgPL3 locus were amplified by PCR with the Phusion polymerase (Thermo Scientific) using primers TgPL3 US F/R and TgPL3 DS F/R (S1 Table). The resulting pBC_mCherry_GFP_305140 vector was linearized prior to electroporation with the BTX ECM 630 Electroporation System. The Q5 mutagenesis kit (NEB) was used to change the Cas9 plasmid (generous gift from Sebastian Lourido) gRNA sequence to 5’-CATTTCCGGGGCAGCGAATC-3’ using primers TgPL3 gRNA F/R to target Cas9 to the AGG PAM site 5’ of the TgPL3 start codon. 100 μg of linearized pBC_mCherry_GFP_305140 and 20 μg of the TgPL3-specific Cas9 plasmid were electroporated into 2 x 10^7^ parasites as described previously [37] and allowed to recover for 2–3 days prior to FACS sorting.

ΔTgPL3::TgPL3 complement strain were created by amplifying cDNA from the TgPL3 coding region using the TgPL3 ORF F/R primers (S1 Table) and inserting the coding region between the endogenous 5’ and 3’ flanking regions used in the knockout construct to allow for homologous recombination of the TgPL3 coding sequence back into its original locus. The ΔTgPL3::TgPL3^S1409A^ PLP domain mutant (S1409A) was created using the Q5 mutagenesis kit and primers Q5 S1409A F/R (S1 Table) to change nucleotide 5969 T>G, resulting in a serine to alanine mutation at amino acid 1409. To target the CGG PAM site within mCherry, the Cas9 plasmid gRNA sequence was changed to 5’-AGGCTGAAGCTGAAGGACGG -3’ using primers mCherry gRNA F/R (S1 Table). Knockout parasites were electroporated as described before with linearized complement constructs and mCherry-specific Cas9 plasmid.

### FACS sorting for clonal populations

The UW Carbone Cancer flow cytometry core assisted us in all FACS cell sorting. The BD FACS AriaII BSL-2 Cell Sorter was kept at room temperature when collecting parasites after sorting. mCherry-expressing RH parasites and GFP-expressing RH parasites are used as gating controls. Each population is scraped and syringed and 1 mL of the extracellular parasites moved into a polystyrene tube for sorting. For the knockout construct, single parasites that expressed mCherry, indicating insertion of the plasmid, but did not express GFP, indicating a successful double crossover event, were sorted directly into a 96-well plate. For the complement constructs, single parasites no longer expressing mCherry were sorted into a 15 mL polystyrene conical and transferred into a fresh flask of HFFs. These populations were then cloned by limiting dilution 2–3 days later.

### Southern blot analysis to confirm knockout and complementation

The neutral transfer method of Southern blot analysis [38] was used to confirm knockout and complementation strains. A probe with homology to the 5’ flank of TgPL3 was amplified by PCR using primers Southern probe F/R. The probe was labeled with [α-32P] dCTP using a random primed DNA labeling kit (Roche). gDNA from all candidate clonal strains was extracted using the TELT method [39] and digested with NotI and PsiI overnight. Briefly, the digested gDNA samples were electrophoresed on an agarose gel, transferred to a positively charged nylon/nytran membrane and exposed to the hybridization probe. After stringent washing, the membrane was positioned against a phosphor screen (Molecular Dynamics) overnight before imaging on a Typhoon FLA 9000 machine (GE).

### Production of TgPL3 antibodies

The TgPL3 PLP domain was expressed using the CellFree Sciences wheat germ system (WEPRO7240). The PLP domain was amplified from cDNA using primers PLP F/R (S1 Table) and inserted into the provided pEU-E01-MCS empty vector, which contains a C-terminal HIS tag. Transcription and translation were performed by manufacturer’s instruction. The resulting protein was purified by nickel column chromatography. Alternatively, three peptides were synthesized (Genscript) from unique locations along the gene based on maximizing the estimated immunogenicity and minimizing cross reactivity (S1 Fig). 100 μg of purified protein or peptide conjugated to KLH was mixed 50:50 with Freund's complete adjuvant and injected subcutaneously into two male BALB/c mice each. Booster intradermal injections of 100 μg of antigen in Freund's incomplete adjuvant were given 4 and 8 weeks later. Serum was collected by cardiac puncture 2 weeks after the final booster. Serum was prepared and stored at -80°C.

### Immunofluorescence

*T*. *gondii*-infected HFF monolayers were fixed in 3.7% formaldehyde in PBS for 20 min, permeabilized and blocked with 0.2% v/v Triton x-100 (Sigma) and 3% BSA in PBS at room temperature for one hour. Primary antibody was incubated at 4°C overnight in 0.2% v/v Triton x-100 and 3% BSA in PBS (1:500 mouse anti-TgPL3, 1:500 rabbit anti-MIC2) washed 3 times with 0.2% Triton in PBS and incubated one hour with the specific secondary antibody (1:1000 goat anti-rabbit Alexa Fluor 488 and 1:1000 goat anti-mouse Alexa Fluor 594) at room temperature for one hour and then washed 3 times with PBS. RH parasites with endogenously tagged CAP1-YFP or RNG1-YFP were also used as colocalization markers. Cells nuclei were stained with DAPI (Sigma). Slides were mounted in Vectashield antifade mounting medium (VectorLabs). Samples were imaged on Zeiss Axioplan III equipped with a triple-pass (DAPI/fluorescein isothiocyanate [FITC]/Texas Red) emission cube, differential interference contrast optics, and a monochromatic Axiocam camera operated by Zen software (Zeiss) and processed using GIMP 2.

### Detergent extraction

Naturally lysed parasites were collected by centrifugation at 1000 x g for 20 minutes at 4°C and resuspended in PBS containing 10 mg/mL deoxycholate (DOC) for 25 minutes at RT. Detergent-extracted parasite samples were settled onto poly-L-lysine coated coverslips for 15 minutes at RT. As a control, untreated parasites were adhered to the poly-L-lysine surface. These samples were fixed with 4% paraformaldehyde for 20 minutes at RT prior to processing for immunofluorescence as described above, except the control slides were stained with IMC7 to indicate disruption of the parasite membrane structures.

### Conoid extrusion

Naturally lysed parasites were pelleted and re-suspended in 20 mM HEPES, 5 mM CaCl_2_ for calcium ionophore and 3 μM A23187. Parasites were then settled onto poly-L-lysine coated coverslips for 15 minutes at 37°C and fixed with 4% paraformaldehyde for 20 minutes at RT prior to processing for immunofluorescence.

### Replication assay

Internal replication assays were performed by seeding HFFs on sterile glass coverslips in 4-well plates (Corning) and infecting the monolayer with 3.2 x 10^4^ parasites in triplicate per strain. The monolayers were washed with PBS to remove external parasites 3–4 hours post infection and fresh media was added to each well. 18 and 26 hours post infection, the monolayers were fixed and stained as above using 1:500 rabbit anti-SAG1 primary antibody and 1:1000 goat anti-rabbit Alexa Fluor 488 secondary antibody. After mounting, the slides were blinded and the number of parasites per vacuole for at least 100 vacuoles were counted per slide.

### Invasion assay

Invasion assays were slightly modified from the previously described red green method [23]. Confluent HFF cells on coverslips in 24 well plates were placed on ice in 300 μL of complete media. Parasites were lysed, spun and resuspended to 5 x 10^6^ per 300 μL of cold complete media. HFFs on ice were infected with 300μL of each parasite strain in triplicate well, then left on ice for 20 min. After the 20 min on ice, parasites were allowed to invade for 5 min at 37°C. Cells were fixed at room temperature by adding 600 μL of 8% paraformaldehyde in HBSS directly in the media, final volume 1.2 mL of 4% paraformaldehyde. After 10 min, the solution was changed and fixation continued for another 10 min. Extracellular parasites were stained with 1:500 rabbit anti-SAG1 primary antibody, then washed 3 times in HBSS. The cells were then permeabilized with 0.2% Triton x-100 and 3% BSA and incubated with either 1:500 mouse chronic serum or 1:500 rat anti-IMC7. The cells were washed three times and incubated with 1:1000 goat anti-rabbit Alexa Fluor 488 and goat anti-mouse or anti-rat Alexa Fluor 594. At least 100 parasites per slide were scored as either red only (internal) or red and green (external). Slides were blinded to reduce bias and 20 random fields were counted per coverslip at the 40X objective.

### Attachment assay

Attachment of all parasite strains was accessed using a modified version of the glutaraldehyde fixed host cell assay [24]. HFF cells were grown to confluency in chamber slides, washed with PBS and fixed in 2% glutaraldehyde/PBS for 5 min at room temperature. Cells were washed 3 times with PBS, then blocked with 0.16M ethanolamine for at 3 days at 4°C. Cells were washed in PBS before adding 200 μl parasites to each chamber at a density of 5 x 10^7^/ml in DMEM/20mM HEPES/3% FBS. Parasites were allowed to attach for 15 min at 37°C, washed 3 times with PBS, and then fixed in 4% formaldehyde/0.02% glutaraldehyde for 20 min. Immunofluorescence was used to detect attached parasites using 1:500 rabbit anti-SAG1 and 1:1000 goat anti-rabbit Alexa Fluor 488. Ten random fields were counted per chamber at the 40X objective (at least 150 parasites per strain per experiment). As a negative control for attachment, all parasite strains were treated with 25 μM BAPTA-AM (Sigma) for 10 min at room temperature.

### Egress

Freshly egressed parasites were pelleted and resuspended to 1 x 10^6^/mL in HFF media. HFF monolayers seeded on coverslips in a 24-well plate were infected with 1 x 10^5^ parasites to a total of 6 wells per strain. After incubation at 37°C for 2 hours, the wells were washed 5 times and allowed to grow another 28 hours. Media was replaced with serum-free media and 3 μM A23187 was added to induce egress in three wells per strain or DMSO was added as a negative control for the remaining three wells per strain. After incubation for 5 minutes at 37°C, parasites were fixed with 4% paraformaldehyde prior to processing for immunofluorescence. Primary antibodies were mouse anti-GRA3 1:100 and rabbit anti-GAP45 1:5000. Secondary antibodies were anti-rabbit Alexa Fluor 594 1:4000 and anti-mouse Alexa Fluor 488 1:4000. At least 200 vacuoles were counted as egressed or not egressed per replicate.

### Western blot analysis of secreted microneme proteins

Microneme secretion assays (Fig 5A and 5B) were slightly modified from the previously described [25]. Parasites were mechanically lysed and resuspended in DMEM plus 20 mM HEPES to a final concentration of 1 x 10^8^/mL. For excreted/secreted antigen (ESA) induction, 200 μL of parasites were added to 200 μL of 2% ethanol plus 6% FBS in DMEM preheated to 37°C. For the control, 200 μL of parasites were added to 200 μL of DMEM only. Both ESA and control were incubated at 37°C for 30 minutes, then chilled on ice for 5 minutes. Tubes were centrifuged at 1000 x g for 10 minutes. The pellet was collected as loading control and the supernatant was centrifuged again. After the second centrifugation, 300 uL of the supernatant was collected and considered the ESA fraction. Pellet and ESA were run on 12% SDS-Page gel and blotted to PVDF membrane. Membranes were blocked in 5% BSA in TBS-T for 3 hours at room temperature, incubated overnight with 1:2000 rabbit anti-MIC2 and 1:4000 monoclonal mouse anti-SAG 1 (DG52) in 5% BSA in TBS-T. Membranes were washed, and incubated with goat anti-rabbit HRP conjugated (Sigma, A0545) and goat anti-mouse HRP conjugated (Invitrogen, 62–6520) for one hour at room temperature in TBS-T. Blots were washed, and incubated with chemiluminescent substrate (Promega, W1001) and imaged using Odyssey FC (LICOR).

For S6C and S6D Fig, 1 x 10^8^ freshly egressed parasites were washed, pelleted and resuspended in intracellular buffer (5 mM NaCl, 142 mM KCl, 1 mM MgCl_2_, 2 mM EGTA, 5.6 mM glucose, 25 mM HEPES pH 7.2). Samples were split into two treatment groups, pelleted and resuspended in serum-free media with either propranolol (500 μM) to induce microneme secretion or DMSO as a negative control. The parasites were incubated at room temperature for 10 minutes, 37°C for 20 minutes and then kept on ice for the remainder of the protocol. Parasites were pelleted, supernatant was transferred to a new tube and the remaining pellet saved. The supernatant was re-pelleted before collecting the final supernatant sample, which contains the excretory secretory antigens (ESA). Pellet and ESA fractions were lysed in laemmli buffer, run on a 10% acrylamide gel and transferred to a PVDF membrane for western blot analysis. The membrane was blocked in PBS-Tween 5% milk, incubated with primary antibody (1:2000 rabbit anti-AMA1 or MIC2) followed by secondary antibody (1:10000 goat anti-rabbit HRP), and revealed with ECL substrate (clarity max, BioRad). The membrane was imaged on a BioRad ChemiDoc.

### E-vacuole rhoptry secretion assay

Freshly egressed parasites were incubated with 1uM Cytochalasin D (Sigma C8273) for 10 minutes. HFF monolayers seeded on coverslips in a 24-well plate were infected with 5 x 10^6^ parasites and incubated for 15 minutes. During this time, the plate was centrifuged for 1 minute at 250 x g and then placed at 37°C before fixing with 4% paraformaldehyde. Samples were processed for immunofluorescence using primary antibodies mouse anti-SAG1 1:2000 and rabbit anti-ROP1 1:3000 and secondary antibodies anti-rabbit 488 1:10000 and anti-mouse 594 1:4000.

### SeCreEt assay for rhoptry secretion

SeCreEt assay protocol was modified from previously published [28]. Toxofilin coupled to Cre-recombinase was inserted into the UPT coding sequence in the ΔTgPL3::TgPL3 and ΔTgPL3::TgPL3^S1409A^ complemented strains. Single guides RNA were generated to cut the 5’ and 3’ end of UPRT gene respectively with primers ML3445/ML3446 and ML2087/ML2088. Amplification of donor DNA was done with KOD polymerase (Promega) using primers ML3522/ML3523. The total population was negatively selected for loss of the UPRT with 5 μM pro-drug 5′-fluo-2′-deoxyuridine (FUDR) and clones were isolated using limiting dilution. Integration at the locus was checked with primers ML3547/ML3190 and ML3546/ML3187.

DsRed cells were seeded in T25 flasks at 2.5 x 10^5^ cells per flask. 24 hours after, the cells were infected with 3 x 10^6^ parasites of the toxofilin-Cre expressing lines, in triplicate, and the media was changed after 1 hour or 6 hours to remove any remaining external parasites. After 24 hours, cells were washed in HBSS and trypsinized before FACS analysis for GFP expression on a LSRFortessa cell analyser (BD Biosciences).

### Mouse infection assays

Parasites were scraped and syringed from HFF monolayers and counted for intraperitoneal injection of 100 or 1000 parental RHΔKu80ΔHPT, ΔTgPL3, complement or S1409A parasite strains. Additionally, mice were injected with 10^4^ or 10^5^ ΔTgPL3 and S1409A parasites. For each independent experiment, NMRI mice were sex-matched and aged 8–10 weeks. Uninfected mice and mice injected with heat-killed parasites were used as controls and each experiment was repeated at least 2 times. Mice were monitored for at least 26 days post infection and surviving mice were checked for seroconversion and brain cysts. Seroconversion was tested by loading tachyzoite lysate on a protein gel and performing western blots using serum from infected mice compared to an uninfected and heat-killed parasite controls. At 26 days post infection, the brains of all surviving mice were harvested, homogenized, processed for immunofluorescence assay using FITC-DBA (1:500) and counted as described previously [11]. Mice infected with ME49 parasites were used as positive immunofluorescence staining controls. Prism Graphpad software was used to analyze survival curve by log-rank (Mantel-Cox).

To measure serum cytokine levels during infection, serum was collected on days 3 and 6 post infection and cytokine levels determined using a cytometric bead array kit (BD) following manufacturer’s instructions. The samples were run on an Attune analysis cytometer (ThermoFisher) at the University of Wisconsin Carbone Cancer Center (Grant P30 CA014520) and analyzed using FlowJo.

To measure parasite dissemination, mice were injected with 1000 parasites and euthanized on day 3 post infection. Spleens and lungs were collected, homogenized and plated by serial dilution on monolayers of HFFs. Plaque forming units per mL of material was calculated based on plaque formation after 8 days.

To test the vaccine potential of ΔTgPL3 parasites, 3 mice were infected with either 1000 ΔTgPL3 or S1409A parasites, or kept uninfected as an age-matched control. 30 days post infection, mice were tested for seropositivity as described above and challenged with 1000 RHΔKu80ΔHPT parasites. Survival, disease symptoms, and weight change were recorded for 18 days post challenge. Disease symptoms were scored on a 1–4 scale, with 1 indicating no pain and distress and 4 indicating pain and distress [40]. Prism Graphpad software was used to analyze survival curve by log-rank (Mantel-Cox).

### Statistical methods

All results are presented at mean values ± SEM. Two-tailed unpaired t-tests were used to determine statistical significance between conditions using GraphPad. In vivo survival curves were analyzed for significance by log-rank (Mantel-Cox) test using GraphPad.

## Supporting information

S1 Fig Antibodies against synthetic TgPL3 peptides confirm localization. **(A)** Three peptides were synthesized (Genscript) from unique locations along the gene based on maximizing the estimated immunogenicity and minimizing cross reactivity. **(B)** One or two female BALB/c mice were immunized with each peptide and serum was collected following administration of multiple boosters. Immunofluorescence of the peptide TgPL3 antibodies (green) co-labeled with MIC2 (red) reproduces localization seen with the wheat germ PLP domain antibody. Images were taken under the same magnification and the scale bar in the lower left corner equals 5 μm.
(TIF)

S2 Fig TgPL3 localizes with ß-tubulin even after DOC extraction. Top five panels show extracellular tachyzoites adhered to poly-L-lysine coated coverslips and stained for immunofluorescence imaging for TgPL3 (green), IMC7 (red) and ß-tubulin (blue). Bottom five panels show TgPL3 localization in extracellular tachyzoites after extraction with deoxycholate extraction (DOC). Images for panels were taken under the same magnification and the scale bars in the lower right corners equal 5 μm.
(TIF)

S3 Fig STM insertional mutant does not disrupt transcription. Northern blot analysis revealed that the original STM mutant had an insertion into the TgPL3 promoter that created a fusion transcript of the chloramphenicol acetyl transferase (CAT) and TgPL3 genes. Transcription is driven by the constitutively active a-tubulin promoter on the pT230-TUB/CAT insertion plasmid. Introns and UTRs are shown in grey and exons are shown in orange.
(TIF)

S4 Fig FACS sorting for clonal populations of ΔTgPL3. RH parasites expressing mCherry or GFP were used as gating controls for RH transfections. Pru parasites expressing either mCherry or GFP were used as gating controls for ME49 transfections. For each transfection, single parasites that expressed mCherry, indicating insertion of the plasmid, but did not express GFP, indicating a successful double crossover event, were sorted directly into a 96-well plate using the BD FACS AriaII BSL-2 Cell Sorter. Parasites positive for both mCherry and GFP indicate a random insertion event. mCherry+/GFP- and mCherry+/GFP+ events are shown as a percentage of the total live, single-cell population from each strain.
(TIF)

S5 Fig TgPL3 is not critical for the replication rate of internalized parasites. Triplicate monolayers of HFFs were infected with 3.4 x 10^4^ *T*. *gondii* parasites. Tachyzoites per vacuole were scored in at least 100 randomly encountered vacuoles per replicate. **(A)** 18 hours post infection, **(B)** 26 hours post infection.
(TIF)

S6 Fig TgPL3 deletion does not affect egress or microneme secretion. **(A)** 9 days post infection, HFF monolayers were stained with crystal violet to reveal *T*. *gondii* plaque formation. Plaques numbers are shown for each strain, shown here the combined results from two independent experiments performed in duplicate. **(B)** HFF monolayers were infected with 1x10^5^ parasites, 6 replicates per strain. 30 hours post infection, cells were treated with DMSO as a negative control in triplicate or egress was induced with A23187 in triplicate for 5 minutes before staining with mouse α-GRA3 and rabbit α-GAP45. At least 200 vacuoles were counted as egressed or not egressed per replicate. **(C)** The excretory secretory antigens (ESA) supernatant fraction was separated from the pellet fraction, which is used as an input control. Microneme secretion was induced with propranolol (ESA Induced) or treated with DMSO as a negative control (ESA Uninduced). Full AMA1 is 63 kDa while the secreted form is 53 kDa. GRA3, the control to ensure the parasites remained intact through processing, is run slightly off the bottom of the gel in this experiment. **(D)** Parasites were processed the same as panel B except the blots were probed for MIC2. Full MIC2 is 115k Da while the secreted form is 95–100 kDa. GRA3 is a control to ensure the parasites remained intact through processing. M is the marker lane using PageRuler (Thermo) where the orange band is 70 kDa. B is a blank lane.
(TIF)

S7 Fig Representative images from the invasion assay. The percentage of parasites that were successfully able to invade the host cell was determined using the red/green invasion assay [21]. Shown here is one of 20 random fields that were counted at the 40X objective. All images were taken at the same magnification and the white scale bar is 20 μm.
(TIF)

S8 Fig Representative attachment assay images. Shown here is one of the ten random fields that were counted for the number of parasites attached to glutaraldehyde fixed host cells at the 40X objective. All images were taken at the same magnification and the white scale bar in lower right corner is 20 μm.
(TIF)

S9 Fig Representative e-vacuoles. Freshly egressed parasites were incubated with Cytochalasin D, then seeded onto HFF monolayers and centrifuged for 1 minute at 250 x g before incubation at 37°C. Parasites were fixed with paraformaldehyde and stained for ROP1 (green) and SAG1 (red). Shown are representative panels of WT parasites with the thin white arrows indicating parasites that have discharged their rhoptry contents and the wide yellow arrowheads indicate parasites that have not discharged their rhoptries. All images were taken at the same magnification and the white scale bar in upper left corner is 5 μm.
(TIF)

S10 Fig FACS plots from SeCreEt assay experiment 1. Toxofilin coupled to Cre-recombinase was inserted into the UPT coding sequence in ΔTgPL3::TgPL3 and ΔTgPL3::TgPL3^S1409A^ complemented strains. DsRed positive cells were infected with 3 x 10^6^ parasites of the toxofilin-Cre expressing lines in triplicate and the media was changed after 1 hour (1Hr) or 6 hours (6Hr). After 24 hours, DsRed cells were dissociated with trypsin and the single-celled suspension was analyzed by Flow Cytometry using a LSRFortessa cell analyser (BD Biosciences). The bar graph shows the number of GFP++ host cells on the y-axis. After 1 hour of invasion, ΔTgPL3 parasites complemented with S1409A showed a highly significant difference in rhoptry secretion compared to parasites complemented with the WT gene (** p<0.01). After 6 hours, this difference was less significant (* p<0.05). Also shown are the FACS plots of for each sample with the DsRed-A channel on the y-axis and the GFP-A channel on the x-axis. The gate used to count the percentage of GFP positives cells was denominated as GFP++. The percentage correspondent to GFP positive cells is indicated for each sample in white.
(TIF)

S11 Fig FACS plots from SeCreEt assay experiment 2. Toxofilin coupled to Cre-recombinase was inserted into the UPT coding sequence in ΔTgPL3::TgPL3 and ΔTgPL3::TgPL3^S1409A^ complemented strains. DsRed positive cells were infected with 3 x 10^6^ parasites of the toxofilin-Cre expressing lines in triplicate and the media was changed after 1 hour (1Hr) or 6 hours (6Hr). After 24 hours, DsRed cells were dissociated with trypsin and the single-celled suspension was analyzed by Flow Cytometry using a LSRFortessa cell analyser (BD Biosciences). The bar graph shows the number of GFP++ host cells on the y-axis. After 1 hour of invasion, ΔTgPL3 parasites complemented with S1409A showed a highly significant difference in rhoptry secretion compared to parasites complemented with the WT gene (** p<0.01). After 6 hours, this difference was not significant (NS). Also shown are the FACS plots of for each sample with the DsRed-A channel on the y-axis and the GFP-A channel on the x-axis. The gate used to count the percentage of GFP positives cells was denominated as GFP++. The percentage correspondent to GFP positive cells is indicated for each sample in white.
(TIF)

S12 Fig ΔTgPL3 and S1409A parasites are avirulent at a low infectious dose. **(A)** Shown is a combination of two independent experiments of 4–5 male NMRI mice each, with a total of 9–10 mice per strain. Mice were infected with 100 *T*. *gondii* parasites and survival was monitored for 28 days post infection. No brain cysts were seen in surviving mice. **(B-E)** Serum was collected from the same mice at 3 days post infection and cytokine levels of **(B)** IFNγ, **(C)** IL6, **(D)** IL12 and **(E)** TNF were determined using a cytokine bead array kit (Biosciences). Shown is a single representative experiment. While one parental-infected mouse did survive, it did not sera convert, have cytokine levels above the uninfected control, or show brain cysts so we removed it from the analysis. * p<0.05, **p <0.01 and ***p<0.001.
(TIF)

S13 Fig Representative infected brain samples. Mice were infected with 1 x 10^5^ ΔTgPL3 (KO) or 2 x 10^3^ ME49 (WT) parasites. Mice were monitored for at least 26 days post infection, then their brains were harvested, homogenized, processed for immunofluorescence assay using FITC-DBA. The left four panels are representative images of mice infected with ΔTgPL3 and the left four panels are representative images of mice infected with of wild type ME49 parasites. All images were taken with the same magnification under the 40X objective, with the white scale bar in the lower right corner equal to 20 μm.
(TIF)

S14 Fig ΔTgPL3 and S1409A parasites are avirulent at a high infectious dose. 3–4 male NMRI mice were infected with 10^4^ or 10^5^ ΔTgPL3 or S1409A parasites only and survival was monitored for 28 days post infection. All mice survived and no brain cysts were seen. Serum collected 28 days post infection and tested for seropositivity with individual strip blots for each mouse (labeled 1, 2, 3 or 4).
(TIF)

S15 Fig ΔTgPL3 and S1409A parasites induce a protective immunity against subsequent infections. 3 male NMRI mice were infected with 1000 ΔTgPL3 or S1409 parasites, or uninfected as a negative control. **(A)** 30 days post infection, mice were tested for seropositivity with individual strip blots for each mouse (labeled 1, 2, or 3). **(B)** After challenged with 1000 RHΔKu80ΔHPT parasites, weight change was recorded for 14 days post challenge and **(C)** disease symptoms were scored on a 1–4 scale, with 1 indicating no pain and distress and 4 indicating pain and distress.
(TIF)

S1 Table Primer list. All primers are listed in the 5’ to 3’ direction with a brief description of their purpose. Primers used in the Gibson assembly (NEB) method include 18–22 bp annealing portions and 18–22 bp overhangs with homology to the backbone vector.
(DOCX)

## References

[1] Flegr J, Prandota J, Sovičková M, Israili ZH. Toxoplasmosis–a global threat. Correlation of latent toxoplasmosis with specific disease burden in a set of 88 countries. PloS one. 2014 3 24;9(3):e90203 10.1371/journal.pone.0090203 24662942 PMC3963851

[2] Dobrowolski JM, Carruthers VB, Sibley LD. Participation of myosin in gliding motility and host cell invasion by *Toxoplasma gondii*. Molecular microbiology. 1997 10;26(1):163–73. 10.1046/j.1365-2958.1997.5671913.x 9383198

[3] Parker ML, Penarete-Vargas DM, Hamilton PT, Guérin A, Dubey JP, Perlman SJ, Spano F, Lebrun M, Boulanger MJ. Dissecting the interface between apicomplexan parasite and host cell: Insights from a divergent AMA–RON2 pair. Proceedings of the National Academy of Sciences. 2016 1 12;113(2):398–403. 10.1073/pnas.1515898113 PMC4720339 26712012

[4] Boothroyd JC, Dubremetz JF. Kiss and spit: the dual roles of *Toxoplasma* rhoptries. Nature Reviews Microbiology. 2008 1;6(1):79 10.1038/nrmicro1800 18059289

[5] Frankel MB, Mordue DG, Knoll LJ. Discovery of parasite virulence genes reveals a unique regulator of chromosome condensation 1 ortholog critical for efficient nuclear trafficking. Proceedings of the National Academy of Sciences. 2007 6 12;104(24):10181–6. 10.1073/pnas.0701893104 PMC1891257 17535896

[6] Senda K, Yoshioka H, Doke N, Kawakita K. A cytosolic phospholipase A2 from potato tissues appears to be patatin. Plant and Cell Physiology. 1996 4 1;37(3):347–53. 10.1093/oxfordjournals.pcp.a028952 8673343

[7] Shewry PR. Tuber storage proteins. Annals of botany. 2003 6 1;91(7):755–69. 10.1093/aob/mcg084 12730067 PMC4242388

[8] Banerji S, Aurass P, Flieger A. The manifold phospholipases A of *Legionella pneumophila*–identification, export, regulation, and their link to bacterial virulence. International Journal of Medical Microbiology. 2008 4 1;298(3–4):169–81. 10.1016/j.ijmm.2007.11.004 18178130

[9] Sawa T, Shimizu M, Moriyama K, Wiener-Kronish JP. Association between *Pseudomonas aeruginosa* type III secretion, antibiotic resistance, and clinical outcome: a review. Critical Care. 2014 12;18(6):668 10.1186/s13054-014-0668-9 25672496 PMC4331484

[10] Wilson SK, Knoll LJ. Patatin‐like phospholipases in microbial infections with emerging roles in fatty acid metabolism and immune regulation by Apicomplexa. Molecular microbiology. 2018 1;107(1):34–46. 10.1111/mmi.13871 29090840 PMC5739999

[11] Mordue DG, Scott‐Weathers CF, Tobin CM, Knoll LJ. A patatin‐like protein protects *Toxoplasma gondii* from degradation in activated macrophages. Molecular microbiology. 2007 1;63(2):482–96. 10.1111/j.1365-2958.2006.05538.x 17166175 PMC3392091

[12] Tobin CM, Knoll LJ. A patatin-like protein protects *Toxoplasma gondii* from degradation in a nitric oxide-dependent manner. Infection and immunity. 2012 1 1;80(1):55–61. 10.1128/IAI.05543-11 22006568 PMC3255658

[13] Magle CT, Pittman KJ, Moser LA, Boldon KM, Knoll LJ. A *Toxoplasma* patatin-like protein changes localization and alters the cytokine response during toxoplasmic encephalitis. Infection and immunity. 2014 2 1;82(2):618–25. 10.1128/IAI.00444-13 24478077 PMC3911373

[14] Lévêque MF, Berry L, Yamaryo‐Botté Y, Nguyen HM, Galera M, Botté CY, Besteiro S. TgPL2, a patatin‐like phospholipase domain‐containing protein, is involved in the maintenance of apicoplast lipids homeostasis in *Toxoplasma*. Molecular microbiology. 2017 7;105(1):158–74. 10.1111/mmi.13694 28419631

[15] Ling L, Goeddel DV. MIP-T3, a novel protein linking tumor necrosis factor receptor-associated factor 3 to the microtubule network. Journal of Biological Chemistry. 2000 8 4;275(31):23852–60. 10.1074/jbc.M001095200 10791955

[16] Wilson P. A., Gardner S. D., Lambie N. M., Commans S. A., and Crowther D. J. Characterization of the human patatin-like phospholipase family. J. Lipid Res. 2006 47: 1940–1949. 10.1194/jlr.M600185-JLR200 16799181

[17] Rydel TJ, Williams JM, Krieger E, Moshiri F, Stallings WC, Brown SM, Pershing JC, Purcell JP, Alibhai MF. The crystal structure, mutagenesis, and activity studies reveal that patatin is a lipid acyl hydrolase with a Ser-Asp catalytic dyad. Biochemistry. 2003 6 10;42(22):6696–708. 10.1021/bi027156r 12779324

[18] Tran JQ, de Leon JC, Li C, Huynh MH, Beatty W, Morrissette NS. RNG1 is a late marker of the apical polar ring in *Toxoplasma gondii*. Cytoskeleton. 2010 9;67(9):586–98. 10.1002/cm.20469 20658557 PMC2998517

[19] Skariah S, Bednarczyk RB, McIntyre MK, Taylor GA, Mordue DG. Discovery of a novel *Toxoplasma gondii* conoid-associated protein important for parasite resistance to reactive nitrogen intermediates. The Journal of Immunology. 2012 4 1;188(7):3404–15. 10.4049/jimmunol.1101425 22387554 PMC3320748

[20] Anderson DM, Schmalzer KM, Sato H, Casey M, Terhune SS, Haas AL, Feix JB, Frank DW. Ubiquitin and Ubiquitin-Modified Proteins Activate the *Pseudomonas aeruginosa* T3SS Cytotoxin, ExoU. Mole Micro. 2011 12 82(6): 1454–1467. 10.1111/j.1365-2958.2011.07904.x PMC3237844 22040088

[21] Sidik SM, Huet D, Ganesan SM, Huynh MH, Wang T, Nasamu AS, Thiru P, Saeij JP, Carruthers VB, Niles JC, Lourido S. A genome-wide CRISPR screen in *toxoplasma* identifies essential apicomplexan genes. Cell. 2016 9 8;166(6):1423–35. 10.1016/j.cell.2016.08.019 27594426 PMC5017925

[22] Huynh MH, Carruthers VB. Tagging of endogenous genes in a *Toxoplasma gondii* strain lacking Ku80. Eukaryotic cell. 2009 4 1;8(4):530–9. 10.1128/EC.00358-08 19218426 PMC2669203

[23] Huynh MH, Rabenau KE, Harper JM, Beatty WL, Sibley LD, Carruthers VB. Rapid invasion of host cells by *Toxoplasma* requires secretion of the MIC2–M2AP adhesive protein complex. The EMBO journal. 2003 5 1;22(9):2082–90. 10.1093/emboj/cdg217 12727875 PMC156089

[24] Mineo JR, Kasper LH. Attachment of *Toxoplasma gondii* to Host Cells Involves Major Surface Protein, SAG-1 (P-30). Experimental Parasit. 1994 8 79(1):11–20. 10.1006/expr.1994.1054 8050521

[25] Huynh M, Boulanger MJ, Carruthers VB. A Conserved Apicomplexan Microneme Protein Contributes to *Toxoplasma gondii* Invasion and Virulence. Infection and Immunity p. 4358–4368 10 2014 Volume 82 Number 10 10.1128/IAI.01877-14 25092910 PMC4187870

[26] Dobrowolski JM, Sibley LD. Toxoplasma Invasion of Mammalian Cells Is Powered by the Actin Cytoskeleton of the Parasite. Cell. 1996 3;84(6):933–939. 10.1016/s0092-8674(00)81071-5 8601316

[27] Hakansson S, Charron AJ, Sibley LD. *Toxoplasma* evacuoles: a two-step process of secretion and fusion forms the parasitophorous vacuole. EMBO J. 2001 6 20(12) 3132–3144. 10.1093/emboj/20.12.3132 11406590 PMC150190

[28] Koshy AA, Fouts AE, Lodoen MB, Alkan O, Blau HM, Boothroyd JC. Toxoplasma secreting Cre recombinase for analysis of host-parasite interactions. *Nat Methods*. 2010 7, 307–309. 10.1038/nmeth.1438 20208532 PMC2850821

[29] Besteiro S, Bertrand-Michel J, Lebrun M, Vial H, Dubremetz JF. Lipidomic analysis of *Toxoplasma gondii* tachyzoites rhoptries: further insights into the role of cholesterol. Biochemical Journal. 2008 10 1;415(1):87–96. 10.1042/BJ20080795 18564055

[30] Del Carmen MG, Mondragon M, Gonzalez S, Mondragon R. Induction and regulation of conoid extrusion in *Toxoplasma gondii*. Cellular microbiology. 2009 6;11(6):967–82. 10.1111/j.1462-5822.2009.01304.x 19416276

[31] Connell E, Darios F, Broersen K, Gatsby N, Peak‐Chew SY, Rickman C, Davletov B. Mechanism of arachidonic acid action on syntaxin–Munc18. EMBO reports. 2007 4 1;8(4):414–9. 10.1038/sj.embor.7400935 17363971 PMC1852766

[32] Jebbari H, Roberts CW, Ferguson DJ, Bluethmann H, Alexander J. A protective role for IL-6 during early infection with *Toxoplasma gondii*. Parasite immunology. 1998 5;20(5):231–9. 10.1046/j.1365-3024.1998.00152.x 9651924

[33] Schlüter D, Kwok LY, Lütjen S, Soltek S, Hoffmann S, Körner H, Deckert M. Both lymphotoxin-α and TNF are crucial for control of *Toxoplasma gondii* in the central nervous system. The Journal of Immunology. 2003 6 15;170(12):6172–82. 10.4049/jimmunol.170.12.6172 12794148

[34] Mueller C, Klages N, Jacot D, Santos JM, Cabrera A, Gilberger TW, Dubremetz JF, Soldati-Favre D. The *Toxoplasma* protein ARO mediates the apical positioning of rhoptry organelles, a prerequisite for host cell invasion. Cell host & microbe. 2013 3 13;13(3):289–301. 23498954 10.1016/j.chom.2013.02.001

[35] Dubey JP. Toxoplasmosis in sheep—the last 20 years. Veterinary parasitology. 2009 7 7;163(1–2):1–4. 10.1016/j.vetpar.2009.02.026 19395175

[36] Martorelli Di Genova B, Wilson SK, Dubey JP, Knoll LJ. Intestinal delta 6-desaturase activity determines host range for *Toxoplasma* sexual reproduction. PLoS Biology. 2019 17(8):e3000364 10.1371/journal.pbio.3000364 31430281 PMC6701743

[37] Soldati D, Boothroyd JC. Transient transfection and expression in the obligate intracellular parasite *Toxoplasma gondii*. Science. 1993 4 16;260(5106):349–52. 10.1126/science.8469986 8469986

[38] Brown T. Southern blotting. Current protocols in molecular biology. 1993 1;21(1):2–9. 10.1002/0471142727.mb0209as21 18265188

[39] Medina-Acosta E, Cross GA. Rapid isolation of DNA from trypanosomatid protozoa using a simple ‘mini-prep’procedure. Molecular and biochemical parasitology. 1993 6 1;59(2):327–9. 10.1016/0166-6851(93)90231-l 8341329

[40] Burkholder T, Foltz C, Karlsson E, Linton CG, Smith JM. Health evaluation of experimental laboratory mice. Current protocols in mouse biology. 2012 6;2(2):145–65. 22822473 10.1002/9780470942390.mo110217 PMC3399545

