# Supplementary figures and images for: A Toxoplasma gondii patatin-like phospholipase contributes to host cell invasion
Source: PLoS Pathog. 2020 Jul 6;16(7):e1008650. doi: 10.1371/journal.ppat.1008650 (PMC7365478; doi:10.1371/journal.ppat.1008650)

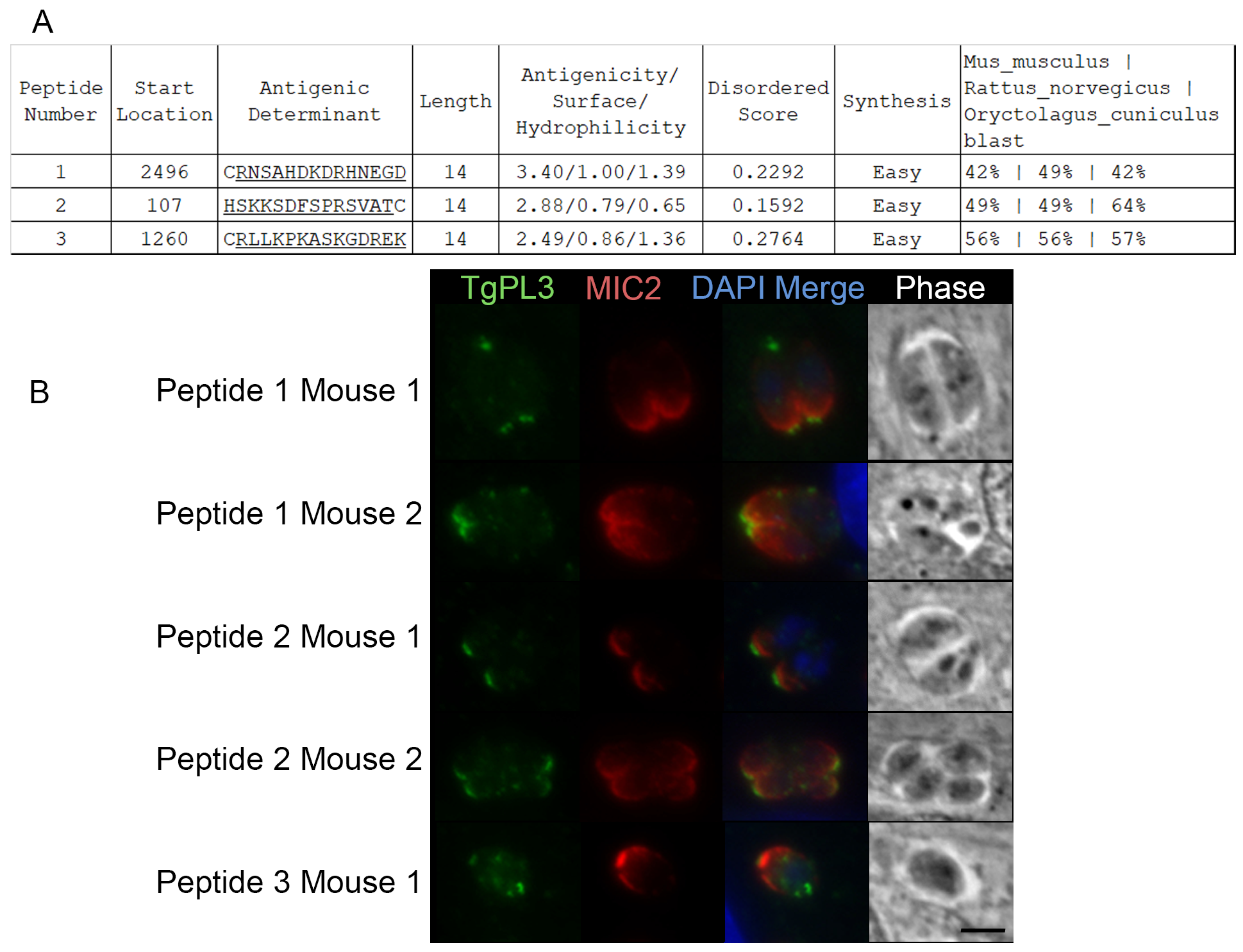

Supplement: S1 Fig — (A) Three peptides were synthesized (Genscript) from unique locations along the gene based on maximizing the estimated immunogenicity and minimizing cross reactivity. (B) One or two female BALB/c mice were immunized with each peptide and serum was collected following administration of multiple boosters. Immunofluorescence of the peptide TgPL3 antibodies (green) co-labeled with MIC2 (red) reproduces localization seen with the wheat germ PLP domain antibody. Images were taken under the same magnification and the scale bar in the lower left corner equals 5 μm. (TIF) [file ppat.1008650.s001.tif]

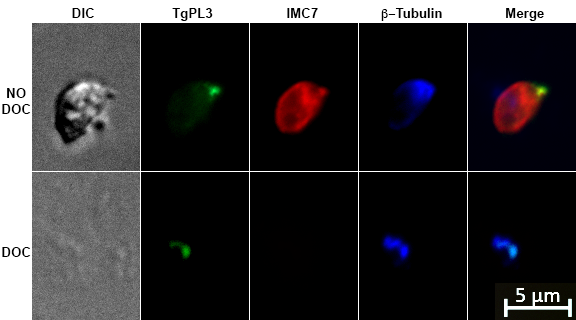

Supplement: S2 Fig — Top five panels show extracellular tachyzoites adhered to poly-L-lysine coated coverslips and stained for immunofluorescence imaging for TgPL3 (green), IMC7 (red) and ß-tubulin (blue). Bottom five panels show TgPL3 localization in extracellular tachyzoites after extraction with deoxycholate extraction (DOC). Images for panels were taken under the same magnification and the scale bars in the lower right corners equal 5 μm. (TIF) [file ppat.1008650.s002.tif]

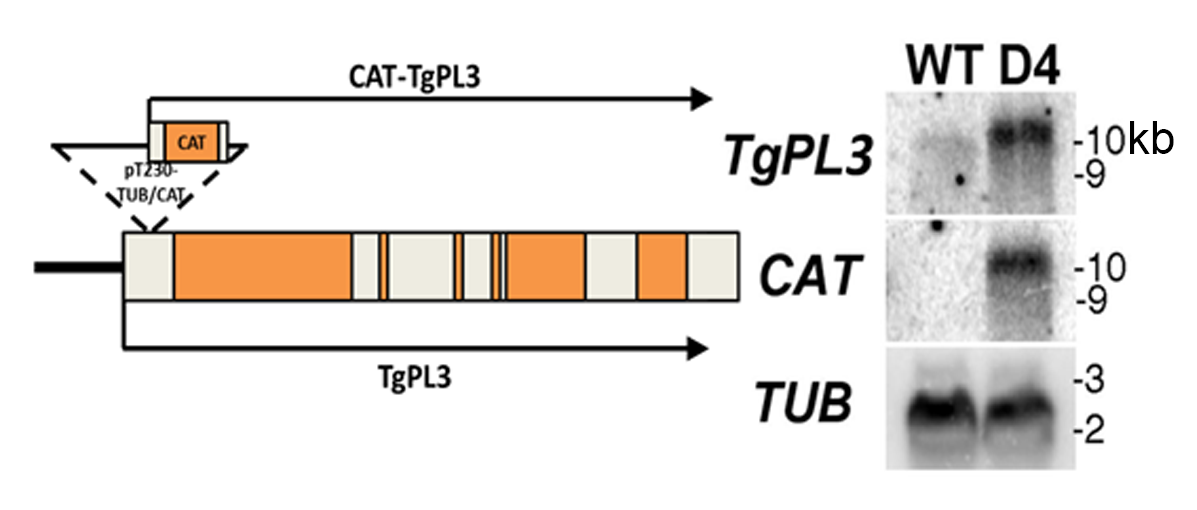

Supplement: S3 Fig — Northern blot analysis revealed that the original STM mutant had an insertion into the TgPL3 promoter that created a fusion transcript of the chloramphenicol acetyl transferase (CAT) and TgPL3 genes. Transcription is driven by the constitutively active a-tubulin promoter on the pT230-TUB/CAT insertion plasmid. Introns and UTRs are shown in grey and exons are shown in orange. (TIF) [file ppat.1008650.s003.tif]

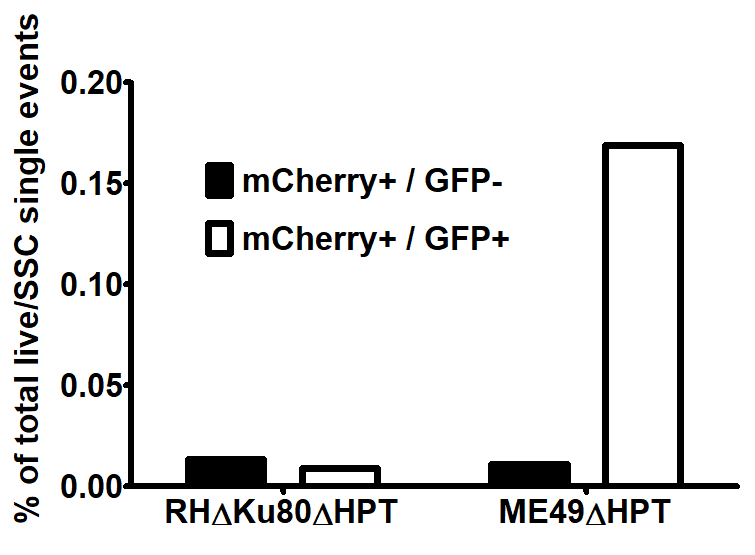

Supplement: S4 Fig — RH parasites expressing mCherry or GFP were used as gating controls for RH transfections. Pru parasites expressing either mCherry or GFP were used as gating controls for ME49 transfections. For each transfection, single parasites that expressed mCherry, indicating insertion of the plasmid, but did not express GFP, indicating a successful double crossover event, were sorted directly into a 96-well plate using the BD FACS AriaII BSL-2 Cell Sorter. Parasites positive for both mCherry and GFP indicate a random insertion event. mCherry+/GFP- and mCherry+/GFP+ events are shown as a percentage of the total live, single-cell population from each strain. (TIF) [file ppat.1008650.s004.tif]

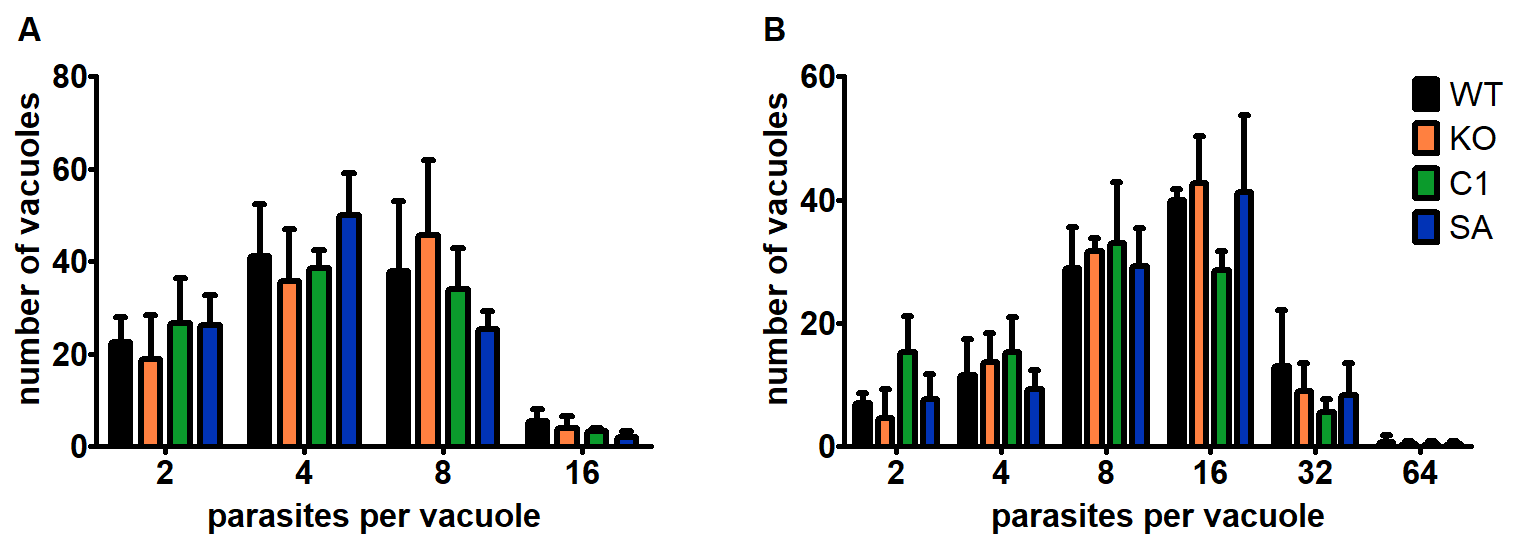

Supplement: S5 Fig — Triplicate monolayers of HFFs were infected with 3.4 x 10^4^ T. gondii parasites. Tachyzoites per vacuole were scored in at least 100 randomly encountered vacuoles per replicate. (A) 18 hours post infection, (B) 26 hours post infection. (TIF) [file ppat.1008650.s005.tif]

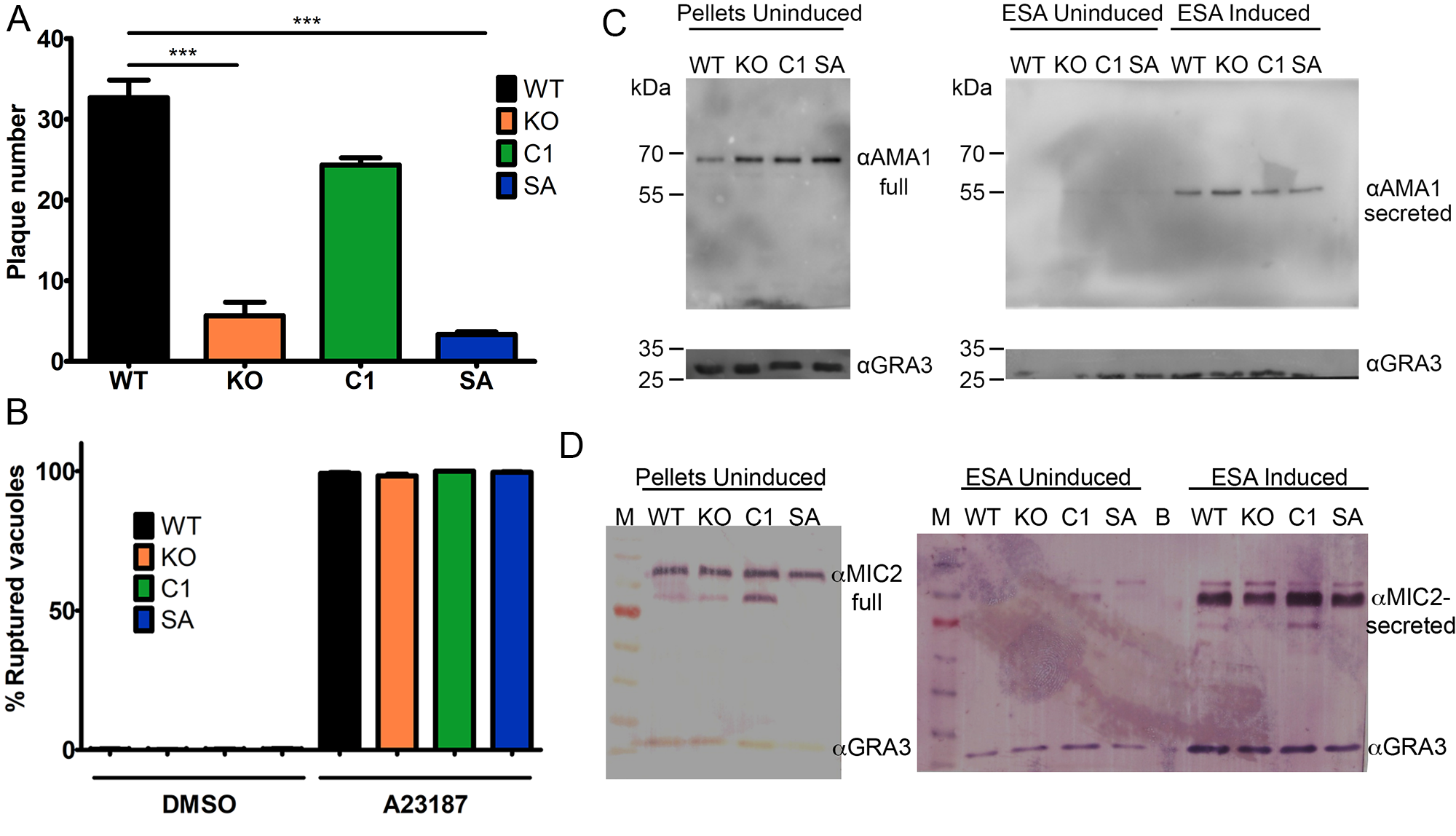

Supplement: S6 Fig — (A) 9 days post infection, HFF monolayers were stained with crystal violet to reveal T. gondii plaque formation. Plaques numbers are shown for each strain, shown here the combined results from two independent experiments performed in duplicate. (B) HFF monolayers were infected with 1x10^5^ parasites, 6 replicates per strain. 30 hours post infection, cells were treated with DMSO as a negative control in triplicate or egress was induced with A23187 in triplicate for 5 minutes before staining with mouse α-GRA3 and rabbit α-GAP45. At least 200 vacuoles were counted as egressed or not egressed per replicate. (C) The excretory secretory antigens (ESA) supernatant fraction was separated from the pellet fraction, which is used as an input control. Microneme secretion was induced with propranolol (ESA Induced) or treated with DMSO as a negative control (ESA Uninduced). Full AMA1 is 63 kDa while the secreted form is 53 kDa. GRA3, the control to ensure the parasites remained intact through processing, is run slightly off the bottom of the gel in this experiment. (D) Parasites were processed the same as panel B except the blots were probed for MIC2. Full MIC2 is 115k Da while the secreted form is 95–100 kDa. GRA3 is a control to ensure the parasites remained intact through processing. M is the marker lane using PageRuler (Thermo) where the orange band is 70 kDa. B is a blank lane. (TIF) [file ppat.1008650.s006.tif]

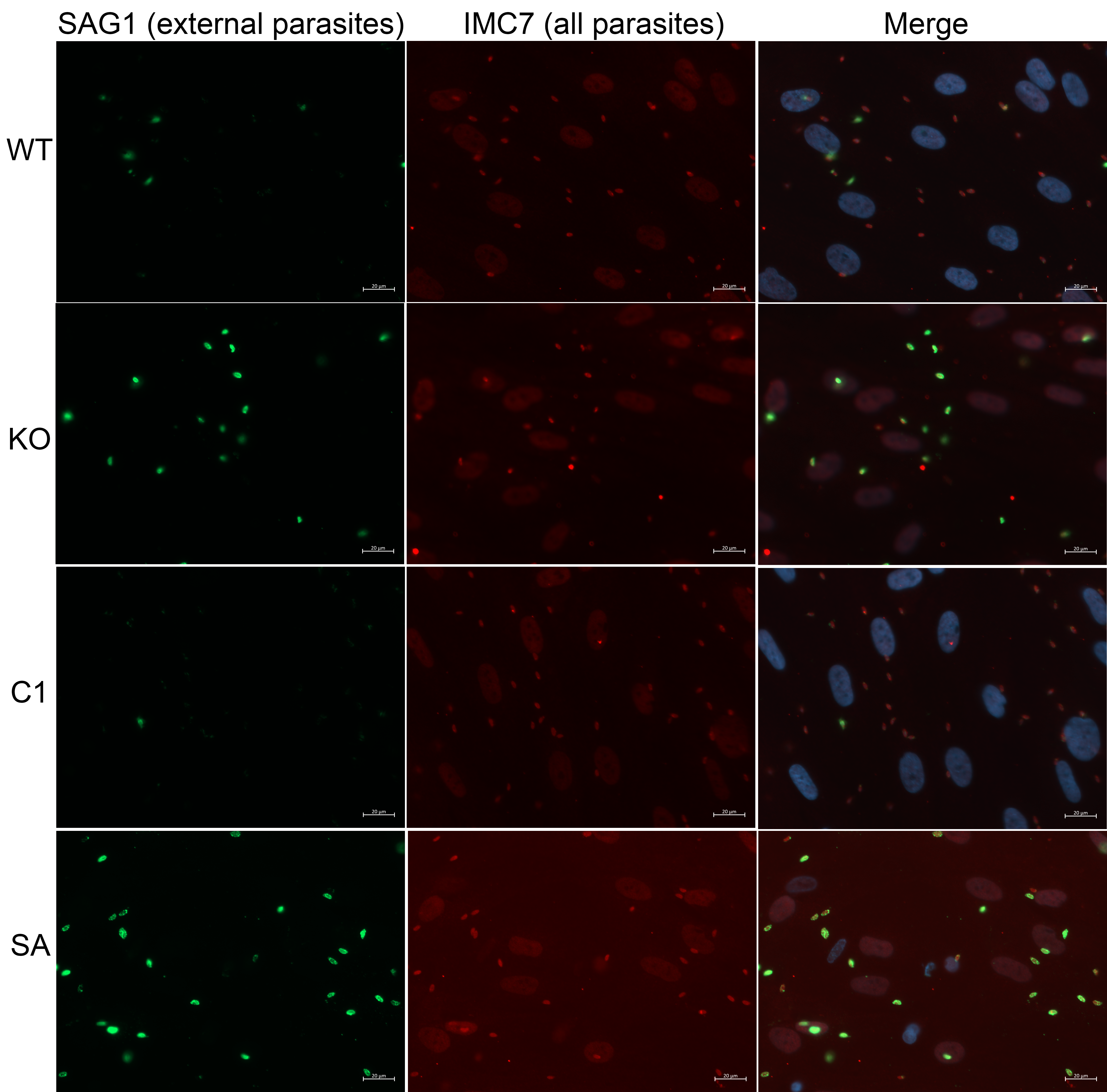

Supplement: S7 Fig — The percentage of parasites that were successfully able to invade the host cell was determined using the red/green invasion assay [21]. Shown here is one of 20 random fields that were counted at the 40X objective. All images were taken at the same magnification and the white scale bar is 20 μm. (TIF) [file ppat.1008650.s007.tif]

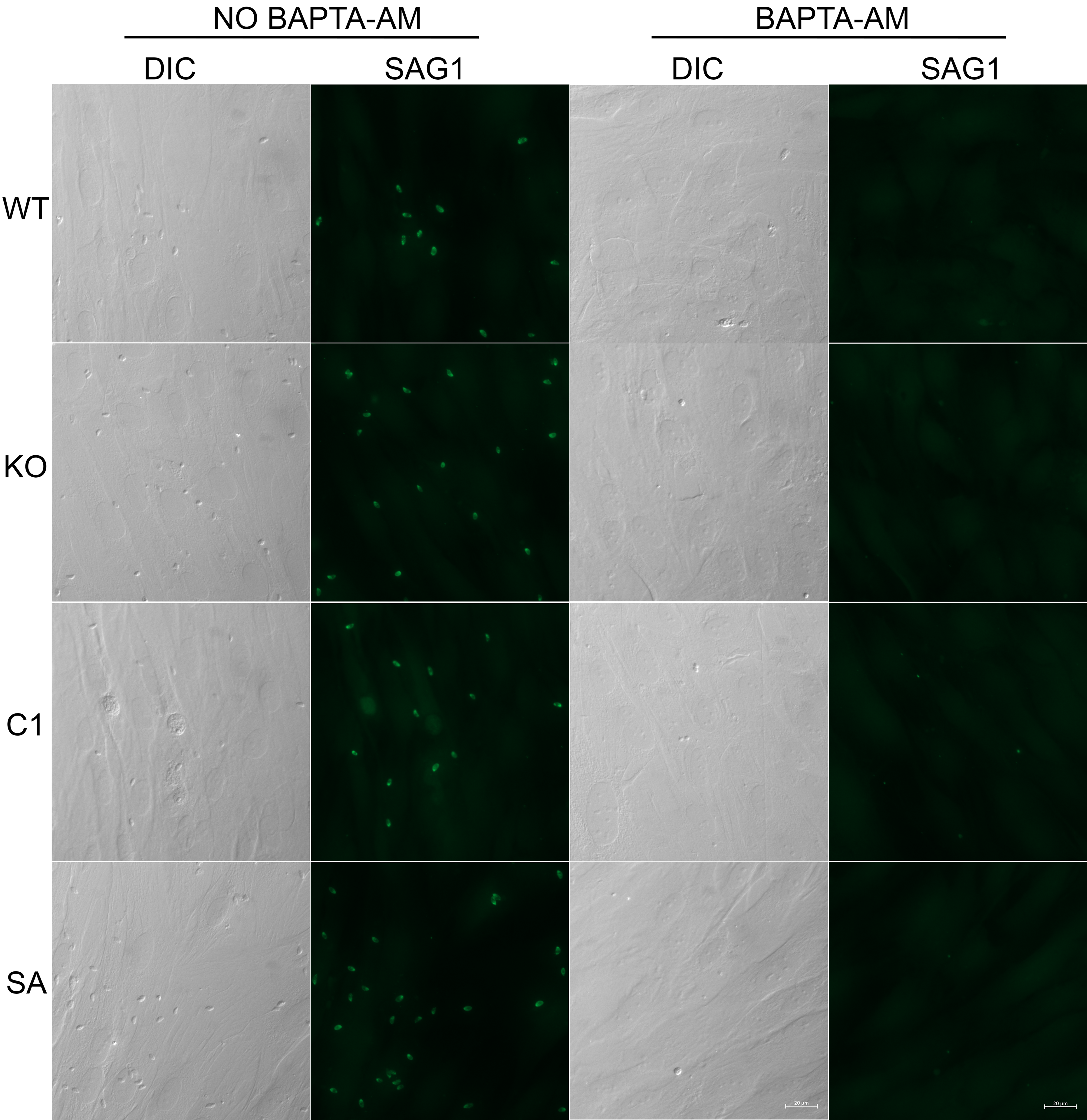

Supplement: S8 Fig — Shown here is one of the ten random fields that were counted for the number of parasites attached to glutaraldehyde fixed host cells at the 40X objective. All images were taken at the same magnification and the white scale bar in lower right corner is 20 μm. (TIF) [file ppat.1008650.s008.tif]

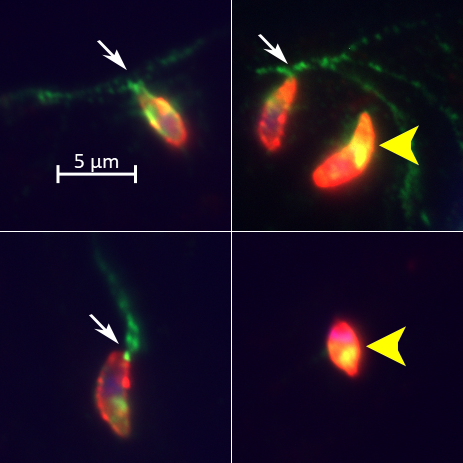

Supplement: S9 Fig — Freshly egressed parasites were incubated with Cytochalasin D, then seeded onto HFF monolayers and centrifuged for 1 minute at 250 x g before incubation at 37°C. Parasites were fixed with paraformaldehyde and stained for ROP1 (green) and SAG1 (red). Shown are representative panels of WT parasites with the thin white arrows indicating parasites that have discharged their rhoptry contents and the wide yellow arrowheads indicate parasites that have not discharged their rhoptries. All images were taken at the same magnification and the white scale bar in upper left corner is 5 μm. (TIF) [file ppat.1008650.s009.tif]

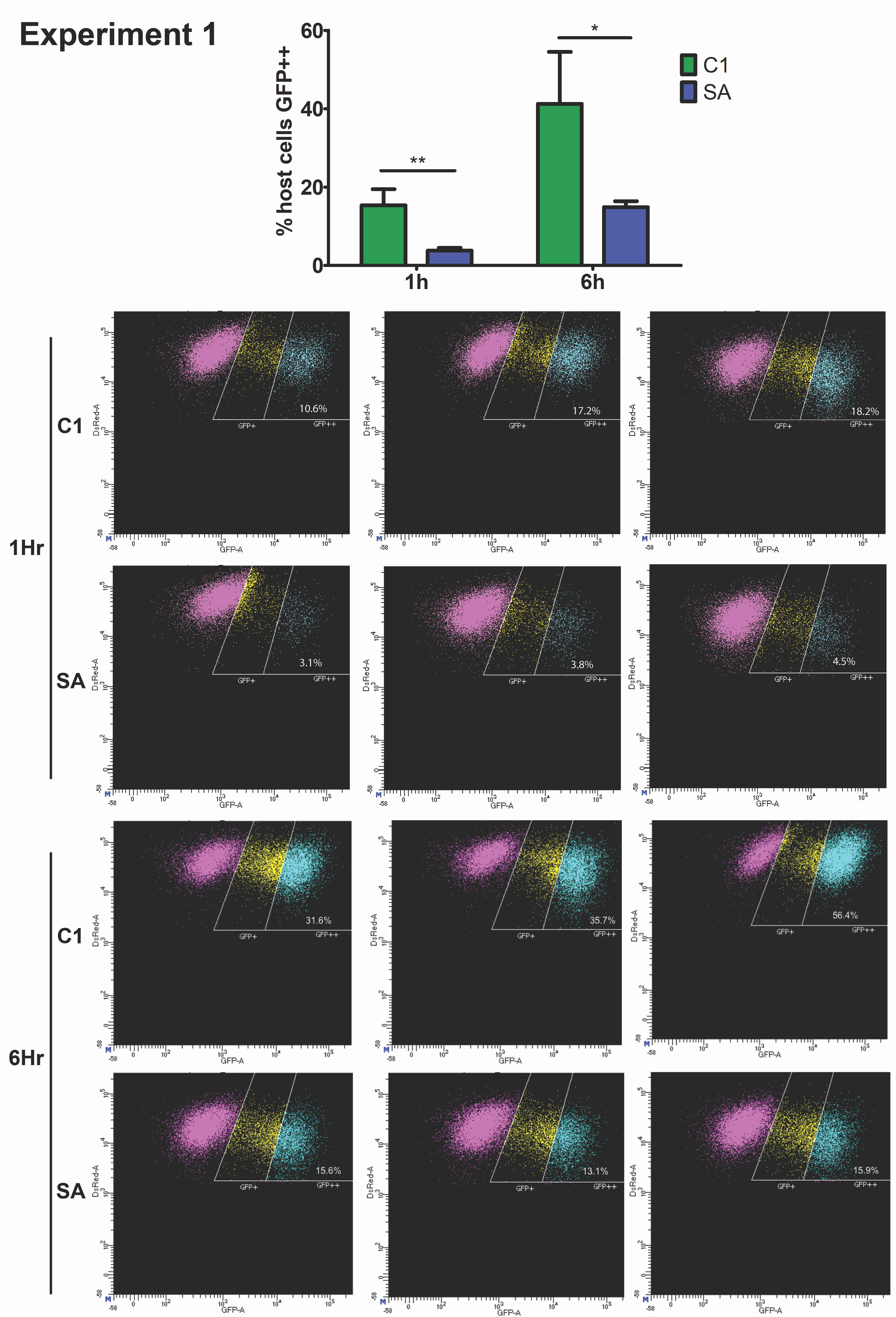

Supplement: S10 Fig — Toxofilin coupled to Cre-recombinase was inserted into the UPT coding sequence in ΔTgPL3::TgPL3 and ΔTgPL3::TgPL3S1409A complemented strains. DsRed positive cells were infected with 3 x 10^6^ parasites of the toxofilin-Cre expressing lines in triplicate and the media was changed after 1 hour (1Hr) or 6 hours (6Hr). After 24 hours, DsRed cells were dissociated with trypsin and the single-celled suspension was analyzed by Flow Cytometry using a LSRFortessa cell analyser (BD Biosciences). The bar graph shows the number of GFP++ host cells on the y-axis. After 1 hour of invasion, ΔTgPL3 parasites complemented with S1409A showed a highly significant difference in rhoptry secretion compared to parasites complemented with the WT gene (** p<0.01). After 6 hours, this difference was less significant (* p<0.05). Also shown are the FACS plots of for each sample with the DsRed-A channel on the y-axis and the GFP-A channel on the x-axis. The gate used to count the percentage of GFP positives cells was denominated as GFP++. The percentage correspondent to GFP positive cells is indicated for each sample in white. (TIF) [file ppat.1008650.s010.tif]

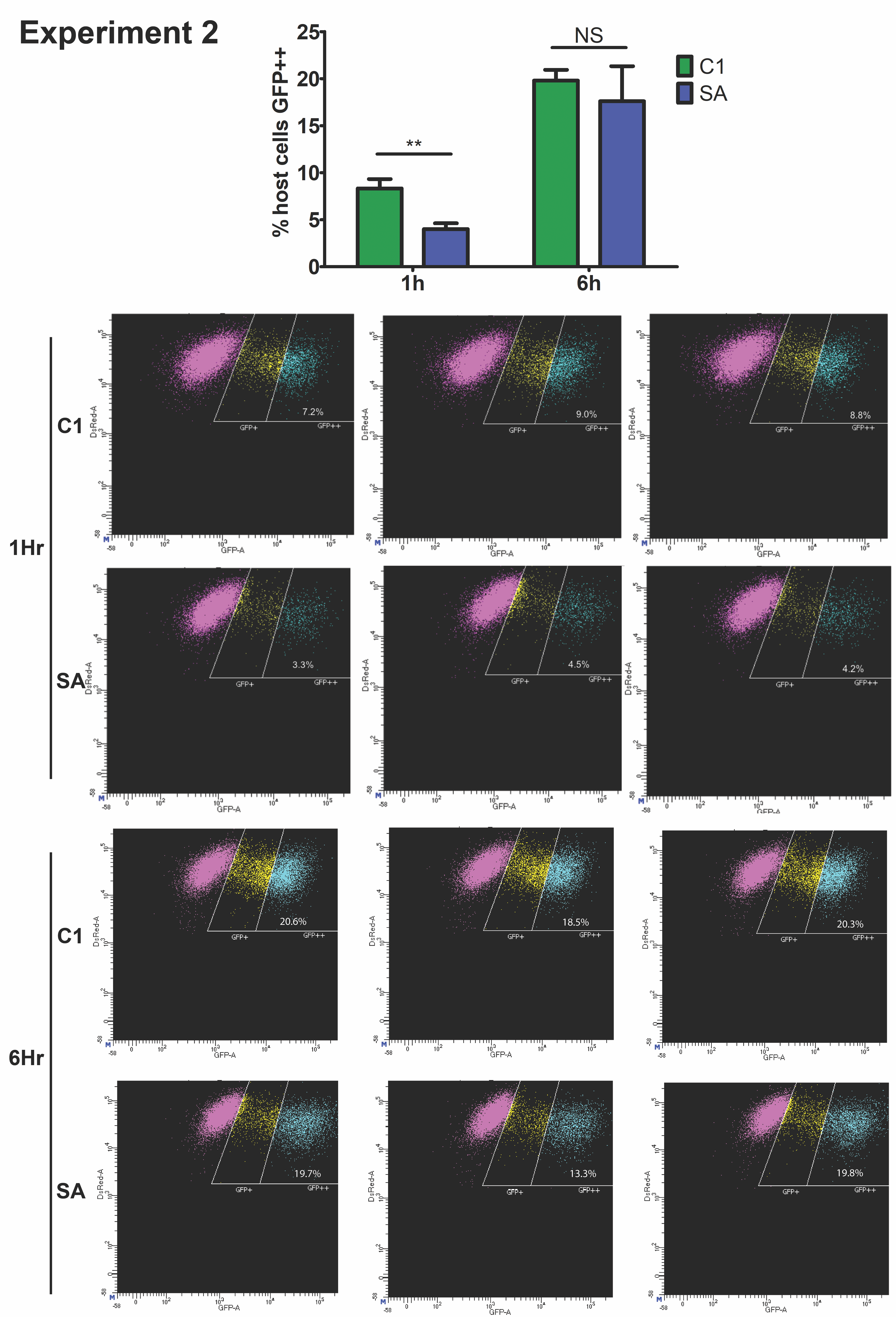

Supplement: S11 Fig — Toxofilin coupled to Cre-recombinase was inserted into the UPT coding sequence in ΔTgPL3::TgPL3 and ΔTgPL3::TgPL3S1409A complemented strains. DsRed positive cells were infected with 3 x 10^6^ parasites of the toxofilin-Cre expressing lines in triplicate and the media was changed after 1 hour (1Hr) or 6 hours (6Hr). After 24 hours, DsRed cells were dissociated with trypsin and the single-celled suspension was analyzed by Flow Cytometry using a LSRFortessa cell analyser (BD Biosciences). The bar graph shows the number of GFP++ host cells on the y-axis. After 1 hour of invasion, ΔTgPL3 parasites complemented with S1409A showed a highly significant difference in rhoptry secretion compared to parasites complemented with the WT gene (** p<0.01). After 6 hours, this difference was not significant (NS). Also shown are the FACS plots of for each sample with the DsRed-A channel on the y-axis and the GFP-A channel on the x-axis. The gate used to count the percentage of GFP positives cells was denominated as GFP++. The percentage correspondent to GFP positive cells is indicated for each sample in white. (TIF) [file ppat.1008650.s011.tif]

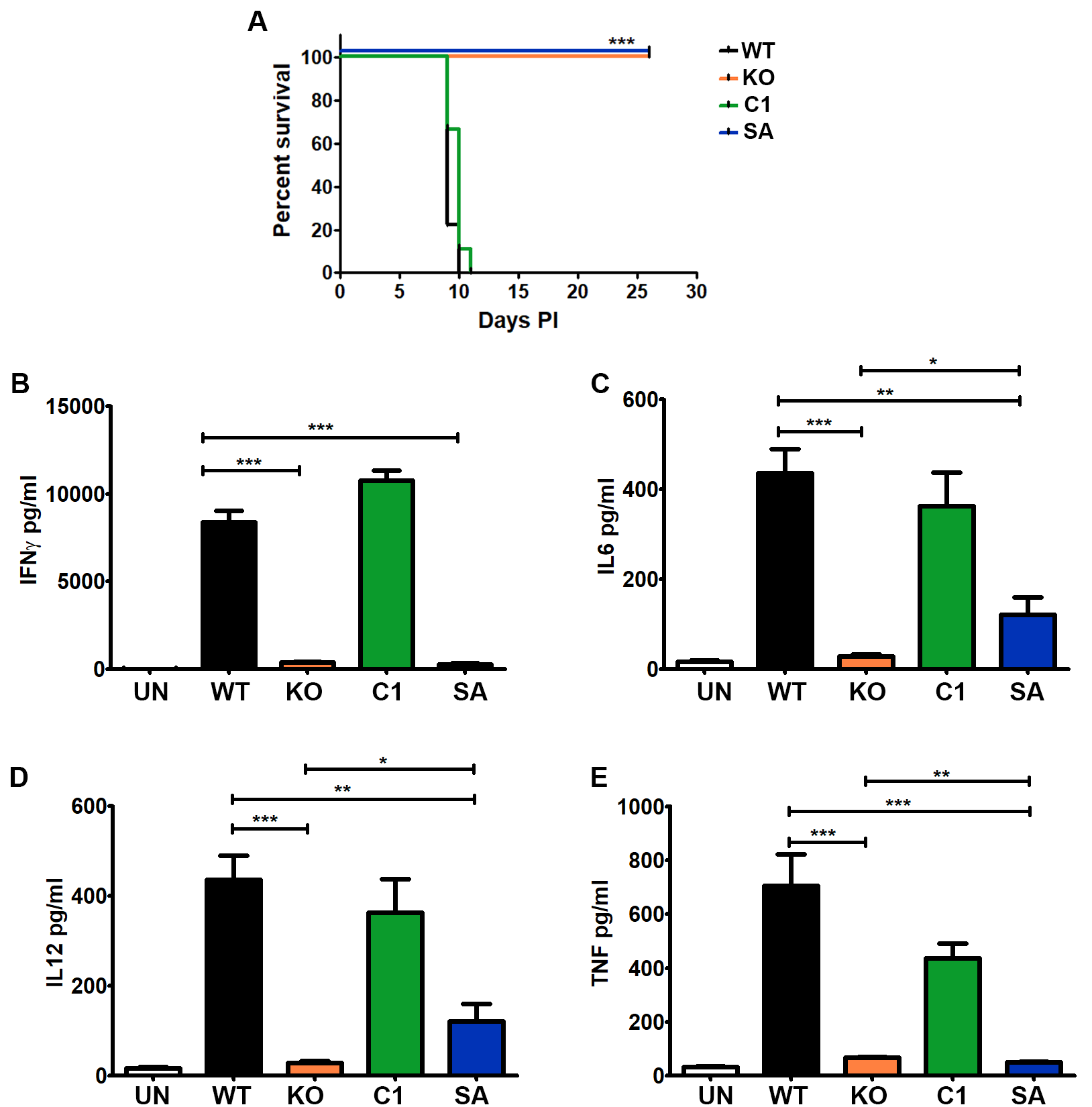

Supplement: S12 Fig — (A) Shown is a combination of two independent experiments of 4–5 male NMRI mice each, with a total of 9–10 mice per strain. Mice were infected with 100 T. gondii parasites and survival was monitored for 28 days post infection. No brain cysts were seen in surviving mice. (B-E) Serum was collected from the same mice at 3 days post infection and cytokine levels of (B) IFNγ, (C) IL6, (D) IL12 and (E) TNF were determined using a cytokine bead array kit (Biosciences). Shown is a single representative experiment. While one parental-infected mouse did survive, it did not sera convert, have cytokine levels above the uninfected control, or show brain cysts so we removed it from the analysis. * p<0.05, **p <0.01 and ***p<0.001. (TIF) [file ppat.1008650.s012.tif]

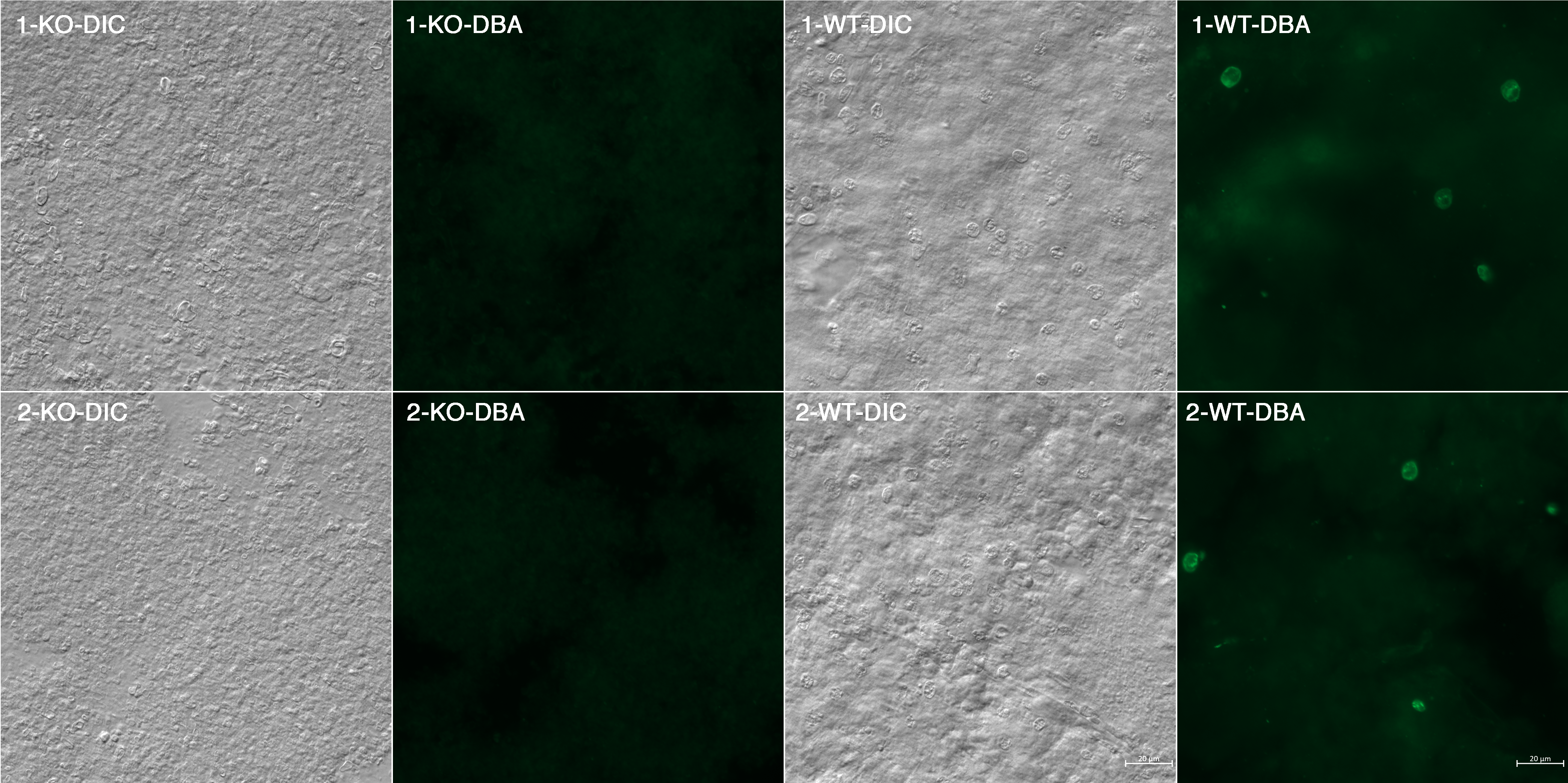

Supplement: S13 Fig — Mice were infected with 1 x 10^5^ ΔTgPL3 (KO) or 2 x 10^3^ ME49 (WT) parasites. Mice were monitored for at least 26 days post infection, then their brains were harvested, homogenized, processed for immunofluorescence assay using FITC-DBA. The left four panels are representative images of mice infected with ΔTgPL3 and the left four panels are representative images of mice infected with of wild type ME49 parasites. All images were taken with the same magnification under the 40X objective, with the white scale bar in the lower right corner equal to 20 μm. (TIF) [file ppat.1008650.s013.tif]

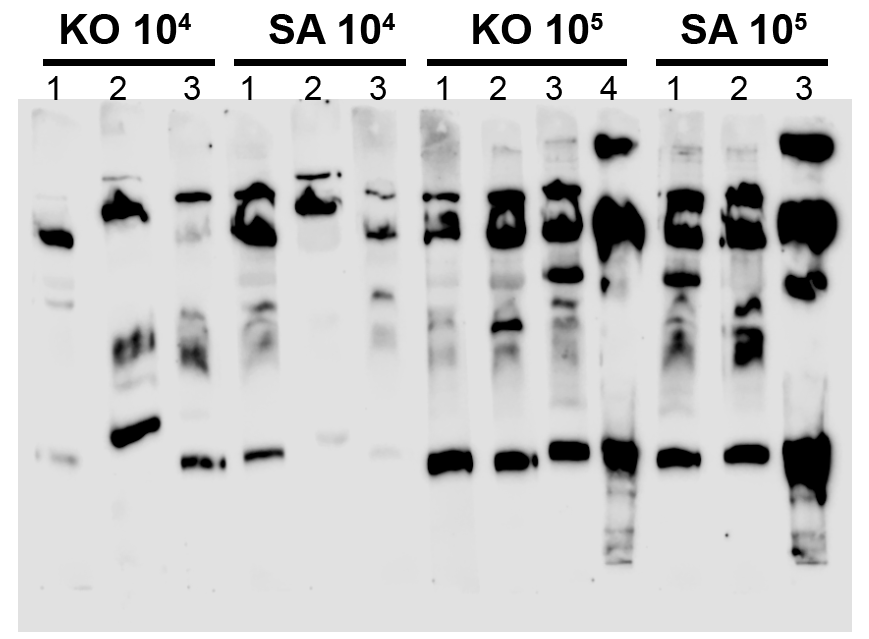

Supplement: S14 Fig — 3–4 male NMRI mice were infected with 104 or 105 ΔTgPL3 or S1409A parasites only and survival was monitored for 28 days post infection. All mice survived and no brain cysts were seen. Serum collected 28 days post infection and tested for seropositivity with individual strip blots for each mouse (labeled 1, 2, 3 or 4). (TIF) [file ppat.1008650.s014.tif]

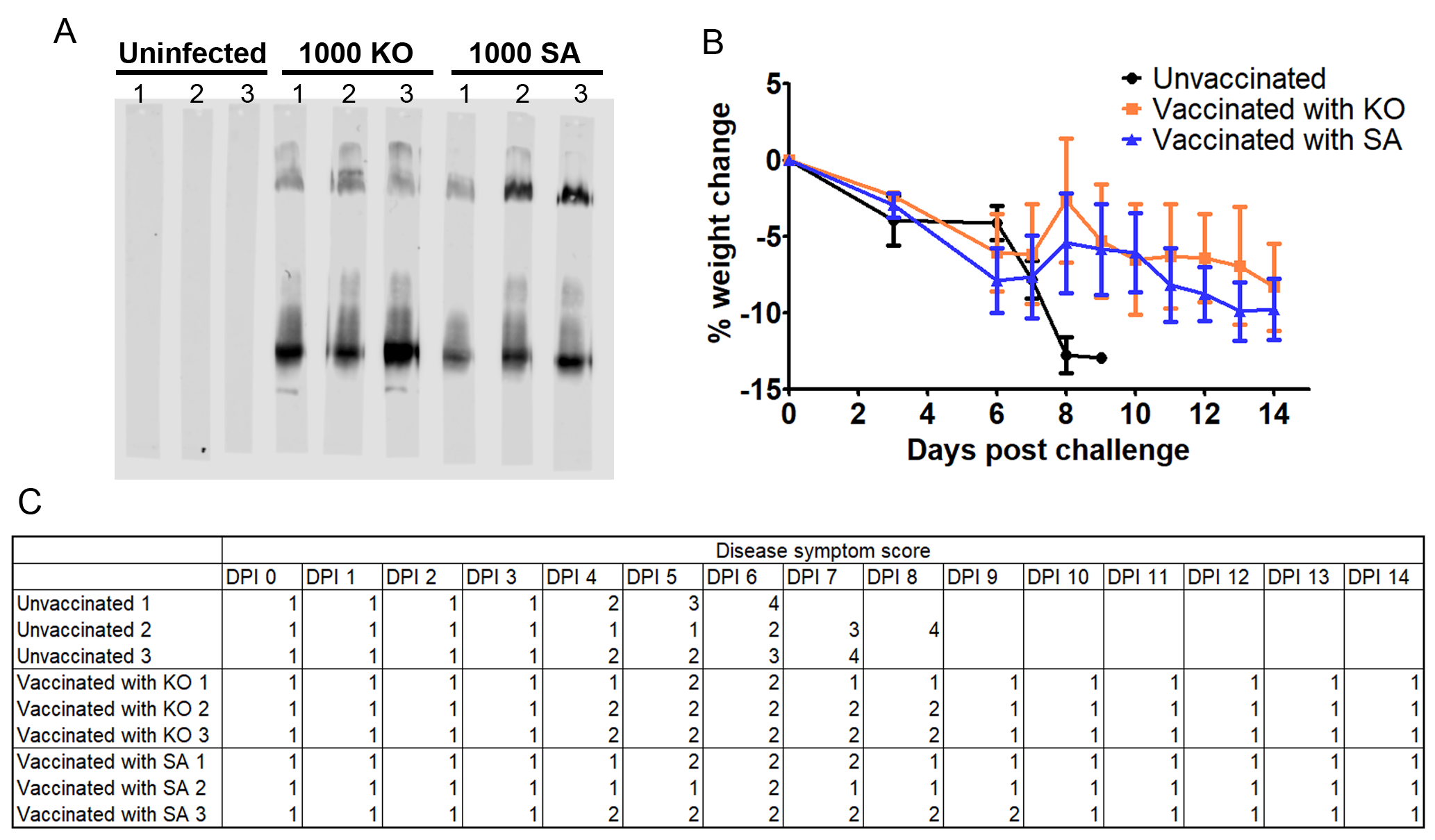

Supplement: S15 Fig — 3 male NMRI mice were infected with 1000 ΔTgPL3 or S1409 parasites, or uninfected as a negative control. (A) 30 days post infection, mice were tested for seropositivity with individual strip blots for each mouse (labeled 1, 2, or 3). (B) After challenged with 1000 RHΔKu80ΔHPT parasites, weight change was recorded for 14 days post challenge and (C) disease symptoms were scored on a 1–4 scale, with 1 indicating no pain and distress and 4 indicating pain and distress. (TIF) [file ppat.1008650.s015.tif]
