# Supplementary material for: A Toxoplasma gondii patatin-like phospholipase contributes to host cell invasion
Source: PLoS Pathog. 2020 Jul 6;16(7):e1008650. doi: 10.1371/journal.ppat.1008650 (PMC7365478; doi:10.1371/journal.ppat.1008650)
Supplement: S1 Table — All primers are listed in the 5’ to 3’ direction with a brief description of their purpose. Primers used in the Gibson assembly (NEB) method include 18–22 bp annealing portions and 18–22 bp overhangs with homology to the backbone vector. (DOCX) [file ppat.1008650.s016.docx]

| Primer name | Use | Primer Sequence (5' to 3') |
| --- | --- | --- |
| GFP GA F | GFP for FACS | AGTGACACTCTAGACTAGTATGTCCGCGTTCGTGAAATTC |
| GFP GA R | GFP for FACS | GCTGGCCTTTTGCTCAACATGTCGGGGGGGCAAGAATTG |
| mCherry GA F | mCherry for FACS | GGGCGAATTGGGTACCTGTCCCGCGTTCGTGAAATTC |
| mCherry GA R | mCherry for FACS | CGGACATACTAGTCTAGAGTGTCACTGTAGCCTGCCAGAAC |
| TgPL3 US F | TgPL3 upstream flank | CTATAGGGCGAATTGGGTACCACGCCTAGTTGCATGCTTG |
| TgPL3 US R | TgPL3 upstream flank | ATTTCACGAACGCGGGACAGCGTGTGATCAGCAACGGAC |
| TgPL3 DS F | TgPL3 downstream flank | GGCTACAGTGACACTCTAGAGAGATGCACCGATGGACG |
| TgPL3 DS R | TgPL3 downstream flank | ACGAACGCGGACATACTAGTACCAAGTGGAAGCCAGTTG |
| TgPL3 gRNA F | TgPL3 gRNA Q5 mutagen. | CATTTCCGGGGCAGCGAATCGTTTTAGAGCTAGAAATAGC |
| TgPL3 gRNA R | TgPL3 gRNA Q5 mutagen. | AACTTGACATCCCCATTTAC |
| TgPL3 ORF F | TgPL3 cDNA complement | CATGGGTTCTCACTCGACGTCGC |
| TgPL3 ORF R | TgPL3 cDNA complement | AATTGGTGCATGCACGTGATGACTC |
| Q5 S1409A F | S1409A Q5 mutagenesis | CGCCGGTGCCGCCGCTGGCGG |
| Q5 S1409A R | S1409A Q5 mutagenesis | ACGGGAGTGTACTGGTTAATCACATTCGCCTCGC |
| mCherry gRNA F | mCherry gRNA Q5 mutagen. | CTGAAGGACGGGTTTTAGAGCTAGAAATAGC |
| mCherry gRNA R | mCherry gRNA Q5 mutagen. | CTTCAGCCTAACTTGACATCCCCATTTAC |
| Southern probe F | Southern blot probe | CCGTTTCTCCCGTTTGTTGG |
| Southern probe R | Southern blot probe | CAGCAACGGACAAGTCTAC |
| PLP F | wheat germ expression PLP | AAAACTAGTGCAAGAAGCGTGACTCTGCG |
| PLP R | mCherry gRNA Q5 mutagen. | AATTCTAGATTAAACCTGCGAACGGTTTTCCTCG |
| ML3522 | SeCreEt donor DNA PCR F | TTGGTCACGCCTCCACAAGGCGTATTCCTCAAAGCACGGAGGAGAGACG |
| ML3523 | SeCreEt donor DNA PCR R | CTTGTCGCGCACCAGGCAGCCTCACAACTATAGGCACCCCAGGCTTTACA |
| ML3445 | SeCreEt gRNA 5’ F | AAGTTGCAGGGCTTCTAAAATGGCGCG |
| ML3446 | SeCreEt gRNA 5’ R | AAAACGCGCCATTTTAGAAGCCCTGCA |
| ML2087 | SeCreEt gRNA 3’ F | AAGTTGGCTCCCACGTCCCTCACCATG |
| ML2088 | SeCreEt gRNA 3’ R | AAAACATGGTGAGGGACGTGGGAGCCA |
| ML3547 | SeCreEt 5’ integration F | GTATCAGTTGTGCGCGGAAG |
| ML3190 | SeCreEt 5’ integration R | TCCCGTTACAGGTGTACGGG |
| ML3546 | SeCreEt 3’ integration F | GAGGGCTAGCAAAGCGTC |
| ML3187 | SeCreEt 3’ integration R | CTCGGCTCCATCTCATTCC |
